# Supplementary material for: Evolution of prosocial punishment in unstructured and structured populations and in the presence of antisocial punishment
Source: PLoS One. 2021 Aug 6;16(8):e0254860. doi: 10.1371/journal.pone.0254860 (PMC8345862; doi:10.1371/journal.pone.0254860)
Supplement: S1 Text — An overview of the models is given. The replicator dynamics of the non-wasteful punishment model is derived. The models are further analyzed and the robustness of the results is argued. Supplementary Videos are explained. (PDF) [file pone.0254860.s001.pdf]

Supplementary Information, S1 Text for:
Evolution of prosocial punishment in unstructured and structured populations and in the presence of antisocial punishment

Mohammad Salahshour

S. 1. OVERVIEW OF THE MODEL

We consider two population structures: a well-mixed population, and a structured population. In the following, we bring the model description for the two different population structures, separately. In addition, we will present an adaptive or non-wasteful punishment model in which if there is nobody to punish, the resources in the punishing pool is redistributed among contributors to the punishing pool.

S. 1.1. Well-mixed population

In the case of a well-mixed population structure, we consider a population of N individuals. At each time step, groups of g individuals are formed at random to play a public goods game (PGG), followed by a public punishing game. In the PGG stage, each individual can either cooperate to defect. Cooperators pay a cost c to invest the same amount c in a public pool. Defectors pay no cost and invest nothing in the public pool. All the investments in the public pool are multiplied by an enhancement factor r and are divided equally among the individuals in the group. After playing the PGG, individuals play a public punishing game. In this stage, cooperators can pay a cost c' to contribute to a social punishing pool, or they can refrain from contributing to the social punishing pool. In the same way, defectors can pay a cost c' to contribute to an antisocial punishing pool, or they can refrain from contributing to the anti-social punishing pool. We call cooperators who contribute to the social punishing pool, punishing cooperators, and those who do not, are called non-punishing cooperators (occasionally both are called cooperators for short). Similarly, defectors who contribute to the antisocial punishing pool are called punishing defectors, and those who do not, are called non-punishing defectors (occasionally both are called defectors for short). All the contributions to a punishing pool are multiplied by an enhancement factor ρ and are spent for punishment purposes. To this end, we assume a fraction α of the resources in the social punishing pool is spent to punish cooperators who do not contribute to the social punishing pool (such that each non-punishing cooperator in a group with n_C non-punishing cooperators and n_{PC} punishing cooperators is punished by an amount $\alpha\rho n_{PC}/n_C)$), and the rest, a fraction $1-\alpha$, is spent to punish (punishing and non-punishing) defectors (such that in a group with n_D non-punishing defectors and n_{PD} punishing defectors, each punishing or non-punishing defector is punished by an amount $(1-\alpha)\rho n_{PC}/(n_D+n_{PD})$)).

Individuals gather payoff from playing the game and reproduce with a probability proportional to the exponential of their payoff, such that the population size remains constant. That is, each individual in the next generation is offspring to an individual in the past generation with a probability proportional to the exponential of its payoff. Offspring inherit the strategies of their parent subject to mutations. We assume mutations in the strategies of the individuals to contribute to the public pool and their strategy to contribute to their respective punishing pool occur independently and with the same probability ν. That is, in each reproduction, with probability ν the offspring of a cooperator (defector) becomes a defector (cooperator), and with the same probability ν, the offspring of a punishing (non-punishing) individual becomes non-punishing (punishing).

S. 1.2. Structured population

In the case of a structured population, we assume that individuals live on a network. For most of the simulations, we assume the population network is a first nearest neighbor, two dimensional square lattice with Moore connectivity and periodic boundaries. That is, each site is connected to all the 8 sites in its vicinity (We will also consider von Neumann connectivity in which each site is connected to four neighboring sites to its right, left, up, and down). Each individual together with its 8 neighbors form a group. That is a total of N groups in the population. A public goods game followed by a public punishing game is played in each group. That is, each individual belongs to 9 groups, each centered around himself or one of its 8 neighbors, and plays 9 games, one in each group that it belongs to.

The games are played in the same way that was the case for a well-mixed population. That is, in each group, cooperators invest in the public goods game at a cost c to themselves, and defectors do not invest. All the investments

in the public pool are multiplied by an enhancement factor r, and are divided equally among the group members. In addition, cooperators can invest in a social punishing pool, at a cost c' to themselves. Similarly, defectors can invest to an antisocial punishing pool at a cost c' to themselves. All the investments to the social and antisocial punishing pool are multiplied by a punishment enhancement factor ρ, and are used for punishing purposes. As was the case in the well-mixed population, this is done by spending a fraction α of the resources in the social punishing pool to punish cooperators who do not contribute to the social punishing pool, and the rest (a fraction $1-\alpha$ of the resources) is spent to punish defectors. Similarly, a fraction α of the investments in the antisocial punishing pool is spent to punish defectors who do not contribute to the punishing pool, and the rest, a fraction $1-\alpha$, is spent to punish cooperators.

After gathering payoff from the games, individuals reproduce with a probability proportional to the exponential of their payoff. For the reproduction, we consider a synchronous update of the network, in which the whole population is updated at the same time. In addition, we consider a *death-birth* update rule. In this update rule, a new individual at each site is an offspring to an individual living in the extended neighborhood of that site. The extended neighborhood of a site is composed of the focal site, together with its neighboring sites. Mutations can occur as well. Similarly to the case of a well mixed population, we assume mutations in the decisions of the individuals to contribute to the public pool, and their decision to contribute to the punishing pool occur independently, each with probability ν. That is, with probability ν the offspring of a cooperator (defector) becomes a defector (cooperator), and with probability ν, a punishing (non-punishing) individual become non-punishing (punishing).

We note that, this dynamics can be considered as an imitation with mutation process as well, in which each individual imitates the strategy of one of the individuals in its extended neighborhood, with a probability proportional to the exponential of their payoff, subject to mutations.

S. 1.3. The non-wasteful punishment model

In the model presented in the main text, if no non-punishing cooperators exist in a group, a fraction α of the resources in the social punishing pool is wasted and is not used for other purposes. Similarly, if no defectors exist in a group, a fraction $1-\alpha$ of the resources in the social punishing pool is wasted. The same is true for the resources in the anti-social punishing pool. That is, if no non-punishing defector exist in a group, a fraction α of the resources in the anti-social punishing pool is wasted, and if no cooperators exist in a group, a fraction $1-\alpha$ of the resources in the anti-social punishing pool is wasted. In a more realistic, and at the same time a more complex model, such unused resources could be used for other purposes. One can expect such adaptivity in spending the resources in the punishing pool helps the functioning of the punishing institution, and thus, helps the evolution of cooperation. To see this is indeed the case, here we consider a simple such adaptive or non-wasteful punishment model. In this model, if there is nobody to punish, the resources in the punishment pool are divided among the contributors to the punishment pool. More precisely, in the non-wasteful punishment model, a fraction α of the resources in the social punishment pool is spent to punish non-punishing cooperators. However, if there is no non-punishing cooperator in the group, instead of being wasted, this is divided equally among the contributors to the social punishing pool (i.e. among the punishing cooperators). Similarly, a fraction $1-\alpha$ of the resources in the social punishing pool is spent to punish defectors. However, if there is no defector to punish in a group, this fraction is divided equally among the contributors to the social punishing pool. The same holds for the anti-social punishing pool. That is, a fraction α of the resources in the anti-social punishing pool is spent to punish non-punishing defectors. However, if there is no non-punishing defector in the group, this is divided equally among the contributors to the anti-social punishing pool (i.e. among the punishing cooperators). Similarly, a fraction $1-\alpha$ of the resources in the anti-social punishment pool is spent to punish cooperators. However, if there is no cooperator to punish in a group, this fraction is divided equally among the contributors to the anti-social punishment pool.

All the other details in this model are as before. That is, individuals gather payoff from the game and reproduce with a probability proportional to the exponential of their payoff, such that the population size remains constant. Each individual in the next generation is offspring to an individual in the past generation with a probability proportional to the exponential of its payoff. Offspring inherit the strategies of their parent subject to mutations. Mutations in the decision of the individuals to contribute to the public pool, and their decision to contribute to the punishing pool occur independently, each with probability ν.

S. 2. REPLICATOR DYNAMICS FOR THE NON-WASTEFUL PUNISHMENT MODEL

The replicator dynamics for the non-wasteful model can be written using similar argument to that used for the previous model. To do this, we begin by the replicator-mutation equation in the general case:

$$m_x(t+1) = \sum_{x'} \nu_x^{x'} m_{x'}(t) \frac{w_{x'}(t)}{\sum_{x''} m_{x''}(t) w_{x''}(t)}. \tag{S.1}$$

Here, the same notation as before is used. For completeness we explain the notation here. x, x', and x'' refer to the strategies, and can be PC, C, PD, or D, referring to, respectively, punishing cooperators, non-punishing cooperators, punishing defectors, and non-punishing defectors. m_x is the frequency of the strategy x, w_x is the expected fitness of an individual with strategy x, and $\nu_x^{x'}$ is the mutation rate from the strategy x' to the strategy x. Under our assumption that mutations in the strategies of the individuals in the public good pool, and in the public punishing pool occur independently, these can be written in terms of the probability of mutation ν, as follows. For those transformations which require no mutations, that is $x = x'$, we have $\nu_x^{x'} = 1 - 2\nu + \nu^2$ (this is the probability that no mutation, neither in the strategy to contribute to the public pool, nor in the strategy to contribute to the punishing pool, occurs). For those rates which require two mutations, one in the decision of the individuals to contribute to the public pool, and one in their decision to contribute to the punishing pool, we have $\nu_D^{PC} = \nu_C^{PD} = \nu_{PC}^{D} = \nu_{PD}^{C} = \nu^2$. All the other rates, which require only one mutation, are equal to $\nu_C^D = \nu_D^C = \nu_{PC}^{PD} = \nu_{PD}^{PC} = \nu_C^{PC} = \nu_{PC}^{C} = \nu_{PD}^{D} = \nu_D^{PD} = \nu - \nu^2$.

To proceed, we need expressions for the expected fitness of different strategies. These are given by the following equations:

$$\begin{aligned}
w_{PC} = & \sum_{n_{PD}=0}^{g-1-n_{PC}-n_C} \sum_{n_C=0}^{g-1-n_{PC}} \sum_{n_{PC}=0}^{g-1} \\
& \exp\left[cr\frac{1+n_C+n_{PC}}{g} - (1-\alpha)c'\rho\frac{n_{PD}}{1+n_{PC}+n_C} + \delta_{n_C,0}\alpha c'\rho + \delta_{n_{PD}+n_D,0}(1-\alpha)c'\rho - c - c'\right] \\
& {\rho_{PC}}^{n_{PC}} {\rho_C}^{n_C} {\rho_{PD}}^{n_{PD}} {\rho_D}^{g-1-n_{PC}-n_C-n_{PD}} \binom{g-1}{n_{PC}, n_C, n_{PD}, g-1-n_{PC}-n_C-n_{PD}}, \\
w_C = & \sum_{n_{PD}=0}^{g-1-n_{PC}-n_C} \sum_{n_C=0}^{g-1-n_{PC}} \sum_{n_{PC}=0}^{g-1} \\
& \exp\left[cr\frac{1+n_C+n_{PC}}{g} - (1-\alpha)c'\rho\frac{n_{PD}}{1+n_{PC}+n_C} - \alpha c'\rho\frac{n_{PC}}{1+n_C} - c\right] \\
& {\rho_{PC}}^{n_{PC}} {\rho_C}^{n_C} {\rho_{PD}}^{n_{PD}} {\rho_D}^{g-1-n_{PC}-n_C-n_{PD}} \binom{g-1}{n_{PC}, n_C, n_{PD}, g-1-n_{PC}-n_C-n_{PD}}, \\
w_{PD} = & \sum_{n_{PD}=0}^{g-1-n_{PC}-n_C} \sum_{n_C=0}^{g-1-n_{PC}} \sum_{n_{PC}=0}^{g-1} \\
& \exp\left[cr\frac{n_C+n_{PC}}{g} - (1-\alpha)c'\rho\frac{n_{PC}}{1+n_{PD}+n_D} + \delta_{n_D,0}\alpha c'\rho + \delta_{n_{PC}+n_C,0}(1-\alpha)c'\rho - c'\right] \\
& {\rho_{PC}}^{n_{PC}} {\rho_C}^{n_C} {\rho_{PD}}^{n_{PD}} {\rho_D}^{g-1-n_{PC}-n_C-n_{PD}} \binom{g-1}{n_{PC}, n_C, n_{PD}, g-1-n_{PC}-n_C-n_{PD}}, \\
w_D = & \sum_{n_{PD}=0}^{g-1-n_{PC}-n_C} \sum_{n_C=0}^{g-1-n_{PC}} \sum_{n_{PC}=0}^{g-1} \\
& \exp\left[cr\frac{n_C+n_{PC}}{g} - (1-\alpha)c'\rho\frac{n_{PC}}{1+n_{PD}+n_D} - \alpha c'\rho\frac{n_{PD}}{1+n_D}\right] \\
& {\rho_{PC}}^{n_{PC}} {\rho_C}^{n_C} {\rho_{PD}}^{n_{PD}} {\rho_D}^{g-1-n_{PC}-n_C-n_{PD}} \binom{g-1}{n_{PC}, n_C, n_{PD}, g-1-n_{PC}-n_C-n_{PD}},
\end{aligned} \tag{S.2}$$

Here, the same notation as before is used, and $\delta_{a,b}$, is the delta function which is equal to 1 if $a = b$ and it is zero otherwise. These expressions can be derived using similar arguments to those used for the original model. The only difference between these, and the expressions for the original model appeared in eq. (2) are the third and fourth terms

in the large bracket in the expressions for w_{PC} and w_{PD}. For completeness, we explain how these expressions can be derived. We begin by noting that, the term inside the large brackets give the payoff of a focal individual with a given strategy, in a group where there are n_{PC} punishing cooperators, n_C non-punishing cooperators, n_{PD} punishing defectors, and $n_D = g-1-n_{PC}-n_C-n_{PD}$ non-punishing defectors in the group. We begin by explaining how to derive the terms in the large brackets.

In a group with n_{PC} punishing cooperators, n_C non-punishing cooperators, n_{PD} punishing defectors and $n_D = g-1-n_{PC}-n_C-n_{PD}$ non-punishing defectors, $cr\frac{1+n_{PC}+n_C}{g}-c$ is the payoff of punishing and non-punishing cooperators and $cr\frac{n_{PC}+n_C}{g}$ is the payoff of punishing and non-punishing defectors from the public goods game. These are the first terms in the large bracket in eq. (S.2). A punishing or non-punishing cooperator, who lives in a group with n_{PC} punishing cooperators, n_C non-punishing cooperators, n_{PD} punishing defectors and $n_D = g-1-n_{PC}-n_C-n_{PD}$ non-punishing defectors, receives a punishment from the punishing defectors equal to $(1-\alpha)c'\rho\frac{n_{PD}}{1+n_{PC}+n_C}$. This is the second term in the large bracket in the expressions for w_{PC} and w_C. In addition, non-punishing cooperators are punished by punishing cooperators in their group, by an amount equal to $\alpha c'\rho\frac{n_{PC}}{1+n_C}$. This is the third term in the large bracket in the expression for w_C. In the case of punishing cooperators, if there are no non-punishing cooperator to punish in the group, the fraction α of the resources in the social punishing pool is not used for punishing purposes, and it is instead redistributed among the punishing cooperators. This is the third term in the expression for w_{PC}. Similarly, if there are no (punishing and non-punishing) defectors in the group, the fraction $(1-\alpha)$ of the resources in the social punishing pool is not used for punishing purposes and is redistributed among punishing cooperators. This is the fourth term in the expression for w_{PC}.

In the same way, a punishing or non punishing defector, who lives in a group with n_{PC} punishing cooperators, n_C non-punishing cooperators, n_{PD} punishing defectors, and n_D non-punishing defectors, receives a punishment from the punishing cooperators equal to $(1-\alpha)c'\rho\frac{n_{PC}}{1+n_{PD}+n_D}$. This is the second term in the large bracket in the expressions for w_{PD} and w_D. In addition, non-punishing defectors are punished by punishing defectors in their group, by an amount equal to $\alpha c'\rho\frac{n_{PD}}{1+n_D}$. This is the third term in the large bracket in the expression for w_D. In the case of punishing defectors, if there are no non-punishing defector to punish in the group, the fraction α of the resources in the anti-social punishing pool is not used for punishing purposes, and it is instead redistributed among the punishing defectors. This is the third term in the expression for w_{PD}. Similarly, if there are no (punishing and non-punishing) cooperators in the group, the fraction $(1-\alpha)$ of the resources in the anti-social punishing pool is not used for punishing purposes and is redistributed among punishing defectors. This is the fourth term in the expression for w_{PD}.

Finally, as punishing cooperators contribute to both the public pool and the social punishing pool, they pay a cost of $c+c'$. On the other hand, non-punishing cooperators only pay a cost of c to contribute to the public pool. Similarly, punishing defectors, pay a cost of c' to contribute to the antisocial punishing pool. Non-punishing defectors contribute to none of the pools and pay no cost. As individuals reproduce with a probability proportional to the exponential of their payoff, the expected fitness of a strategy can be defined as the expected value of the exponential of their payoff.

To calculate the expected value of fitness, we note that ${\rho_{PC}}^{n_{PC}}{\rho_C}^{n_C}{\rho_{PD}}^{n_{PD}}{\rho_D}^{g-1-n_{PC}-n_C-n_{PD}}$ $\binom{g-1}{n_{PC},n_C,n_{PD},g-1-n_{PC}-n_C-n_{PD}}$, is the probability that a focal individual finds itself in a group with n_{PC} punishing cooperators, n_C non-punishing cooperators, n_{PD} punishing defectors, and n_D non-punishing defectors. Here, $\binom{g-1}{n_{PC},n_C,n_{PD},g-1-n_{PC}-n_C-n_{PD}} = \frac{(g-1)!}{n_{PC}!,n_C!,n_{PD}!,(g-1-n_{PC}-n_C-n_{PD})!}$ is the multinational coefficient. This is the number of ways that among the $g-1$ group-mates of a focal individual, n_{PC}, n_C, n_{PD}, and $g-1-n_{PC}-n_C-n_{PD}$ individuals are respectively, punishing cooperators, non-punishing cooperators, punishing defectors, and non-punishing defectors. Summation over all the possible configurations gives the expected fitness of different strategies. Using the expressions in eq. (S.2) for the expected fitness of different strategies in eq. (S.1), we have a set of four equations which gives an analytical description of the model, in the limit of infinite population size.

S. 3. ANALYSIS OF THE MODEL: MIXED POPULATION

S. 3.1. The frequency of different strategies

The frequency of different strategies in the $r-\rho$ plane is plotted in Fig. (S.1). In Fig. (S.1.a) to Fig. (S.1.d), numerical solutions of the replicator dynamics are used, and in Fig. (S.1.e) to Fig. (S.1.h), the results of simulations in a population of size $N = 10000$ is presented. Fig. (S.1.a) and Fig. (S.1.e) represent the frequency of punishing cooperators, m_{PC}, Fig. (S.1.b) and Fig. (S.1.f) show the frequency of non-punishing cooperators, m_C, Fig. (S.1.c) and Fig. (S.1.g) show the frequency of punishing defectors, m_{PD}, and Fig. (S.1.d) and Fig. (S.1.h) show the frequency of non-punishing defectors, m_D. For the solution of the replicator dynamics, a homogeneous initial condition in which the frequency of all the strategies equals 0.25 is used, and for the simulations, a random initial condition in which the

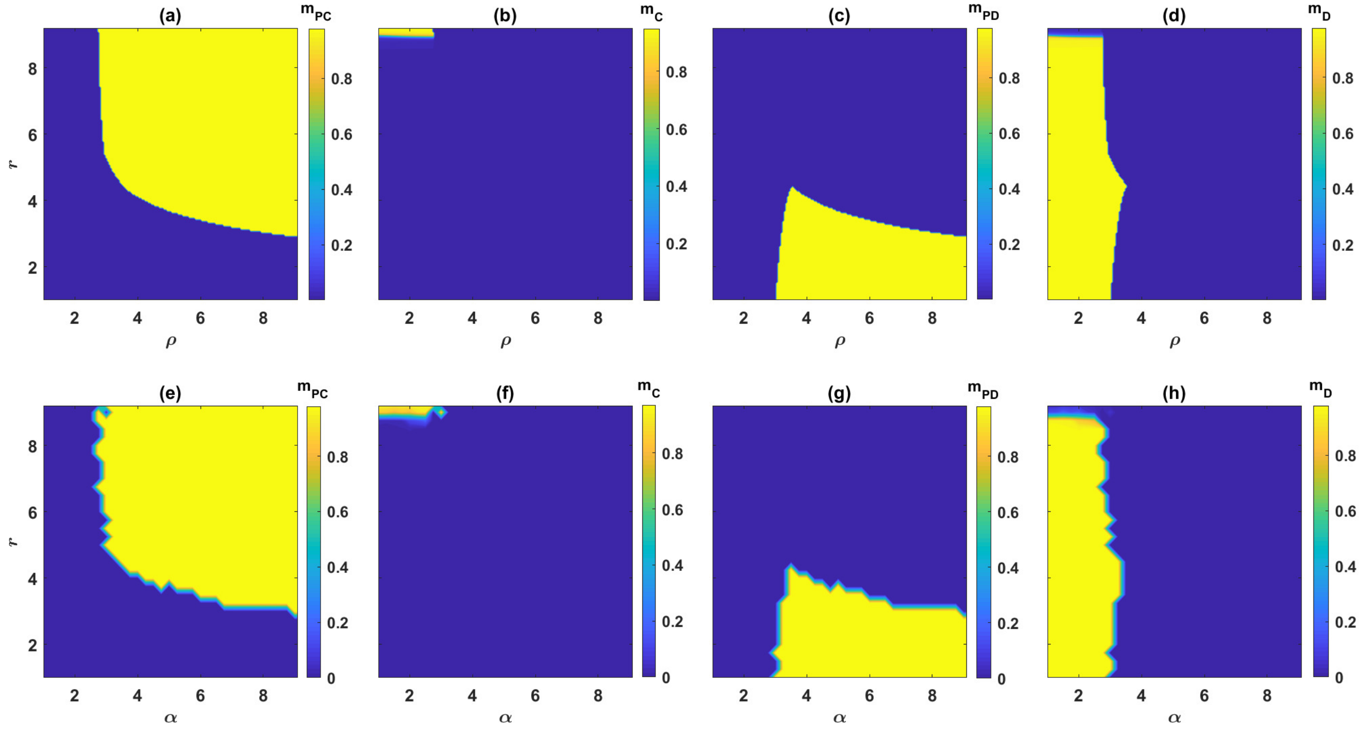

FIG. S.1. Contour plot of the frequency of different strategies in a well-mixed population, in the $\rho - r$ plane, resulted from the replicator dynamics ((a) to (d)), and resulted from simulations in a population of size $N = 10000$ ((e) to (h)). Here, for the solution of the replicator dynamics, a homogeneous initial condition in which the frequency of all the strategies equals 0.25 is used, and for the simulations a random initial condition in which the strategies of the individuals are assigned at random is used. Here, $g = 9$, $\nu = 10^{-3}$, $c = c' = 1$, and $\alpha = 0.5$. The simulations are run for $T = 200$ time steps, and an average over the last 10 steps of the simulations is taken.

strategies of the individuals are assigned at random is used. Here, we have set $g = 9$, $\nu = 10^{-3}$, and $\alpha = 0.5$. The phase diagram presented in Fig. (1.a) in the main text are derived from these results by locating the transition lines and different phases.

As can be seen, the results of the replicator dynamics are in high agreement with the result of simulations. Both the replicator dynamics and the simulations show that the system has four different phases. For r smaller than a value close to $g = 9$, and for small values of ρ, such that the return to the investments in the punishing pool are small, the system goes to a phase where only non-punishing defectors survive. For larger values of ρ, such that the return to the investments in the punishing pool is high enough, punishing strategies evolve. In this region, for smaller values of r, punishing defectors survive and dominate the population. On the other hand, as r increases, the dynamics settle in a phase where punishing cooperators drive other strategies to extinction and dominate the population. The value of r for which the transition between these two phases occurs depends on ρ and decreases with increasing ρ.

The densities of different strategies in the $r - \alpha$ plane are presented in Fig. (S.2). Fig. (S.2.a) to Fig. (S.2.d) represent the numerical solutions of the replicator dynamics, and in Fig. (S.2.e) to Fig. (S.1.h), the result of a simulation in a population of size $N = 10000$ is presented. Fig. (S.2.a) and Fig. (S.2.e) represent m_{PC}, Fig. (S.2.b) and Fig. (S.2.f) represent m_C, Fig. (S.2.c) and Fig. (S.2.g) represent m_{PD}, and Fig. (S.2.d) and Fig. (S.2.h) show m_D. For the solution of the replicator dynamics, a homogeneous initial condition in which the frequency of all the strategies equals 0.25 is used, and for the simulations a random initial condition in which the strategies of the individuals are assigned at random is used. Here, as before, we have set $g = 9$, $\nu = 10^{-3}$, and $\rho = 5$. The phase diagrams presented in Fig. (1.b) in the main text, are derived from these results by locating the transition lines and different phases.

We note that for small α, such that the punishment of second-order free riders is not sufficiently strong, punishment does not evolve: Non-punishing defectors dominate the population as long as the enhancement factor, r, is smaller than a value close to $g = 9$. Punishment evolves, as α increases beyond a threshold. In this region, the punishment of second-order free riders is strong enough to overcome the benefit of second-order free riding. However, whether social or anti-social punishment evolves depends on the enhancement factor of the PGG, r. For large values of r, social punishers dominate the population. On the other hand, for small values of r, antisocial punishment evolves.

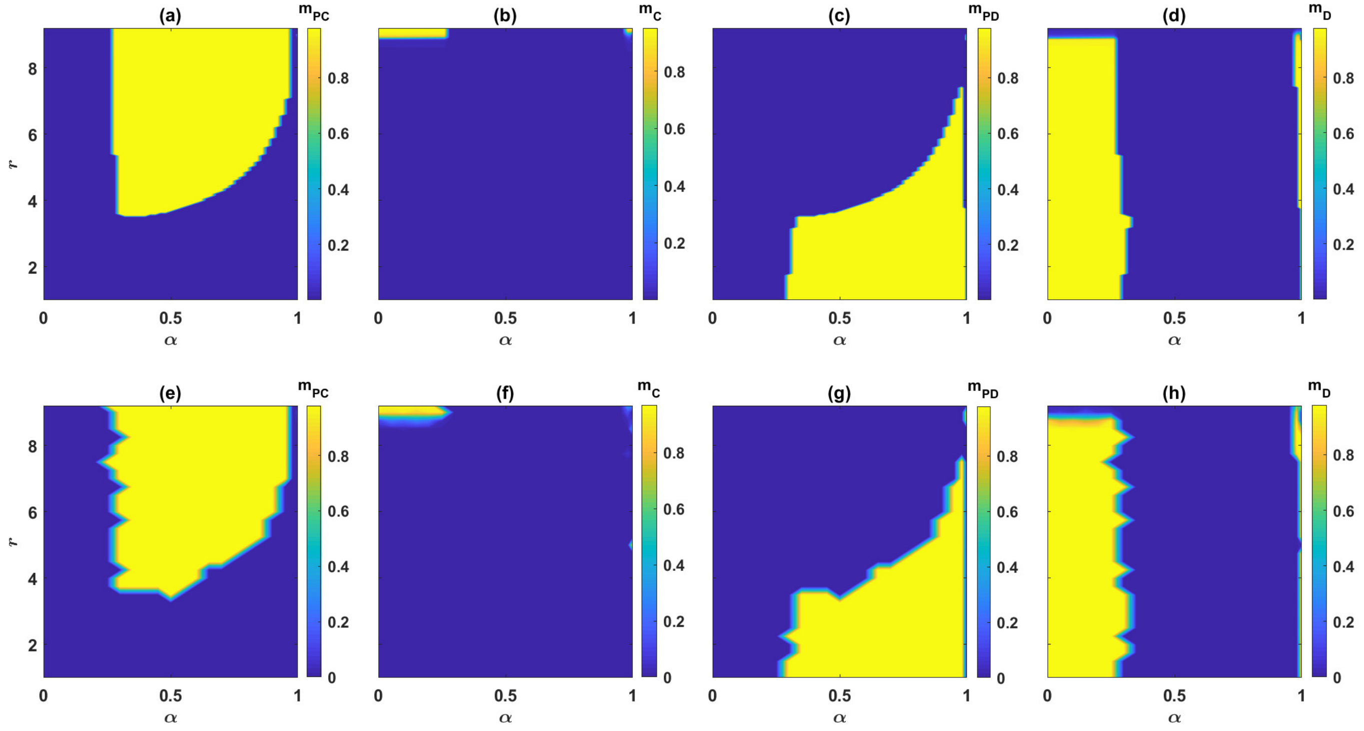

FIG. S.2. Contour plot of the frequency of different strategies in a well-mixed population, in the $\alpha - r$ plane, resulted from the replicator dynamics ((a) to (d)), and resulted from simulations in a population of size $N = 10000$ ((e) to (h)). Here, for the solution of the replicator dynamics, a homogeneous initial condition in which the frequency of all the strategies equals 0.25 is used, and for the simulations a random initial condition in which the strategies of the individuals are assigned at random is used. Here, $g = 9$, $\nu = 10^{-3}$, $c = c' = 1$, and $\rho = 5$. The simulations are run for $T = 200$ time steps, and an average over the last 10 steps of the simulations is taken.

Interestingly, for very large values of α, the evolution of social punishment requires a larger value of r. This shows, an optimal value of α, that is an optimal weight of second-order with respect to first-order punishment exists which facilitates the evolution of social punishment, in the sense that for such an optimal value of α, the transition to the social punishment phase occurs for a smaller value of r.

Finally, for too large values of α, punishing strategies do not evolve, and non-punishing defectors dominate the population. Our analysis thus shows, for social punishment to evolve, it is necessary that both first-order free-riders (those who do not contribute to the public pool), and second-order free-riders (those who do not contribute to the punishing pool) to be punished. Similarly, for antisocial punishment to evolve, it is necessary that both cooperators and non-punishing defectors to be punished.

S. 3.2. The nature of the phase transitions

The system is multistable in the whole region of the phase diagram, and depending on the initial conditions, the dynamics settle into one of the stable phases. For $r < g$, three stable phases exist. PC phase, in which punishing cooperators dominate and drive all the other strategies into extinction, PD phase, in which punishing defectors dominate, and the D phase, in which non-punishing defectors dominate. For $r > g$, PD and D phases become unstable. In this region, two stable phases exist, the C phase, in which non-punishing cooperators dominate, and the PC phase. The multi-stability of the system, implies that the $D - PD$ transition, the $C - PC$ transition, and the $PD - PC$ transition, are discontinuous. The $C - D$ transition shows a different behavior compared to the other transitions. While for large ρ this transition is a discontinuous transition, for small ρ there is cross over from the D phase to the C phase, without passing any singularity. In between, there is a critical point where this transition becomes a continuous transition. This can be seen in Fig. (S.3.a) and Fig. (S.3.b), where the frequency of, respectively, non-punishing cooperators, m_C, and non-punishing defectors, m_D, as a function of r, for three different values of ρ, close to the $C - D$ transition are plotted. Here, the replicator dynamics is solved, setting $g = 9$ and $\nu = 0.001$, and starting from two different initial conditions. As a first initial condition, the initial frequency of non-punishing

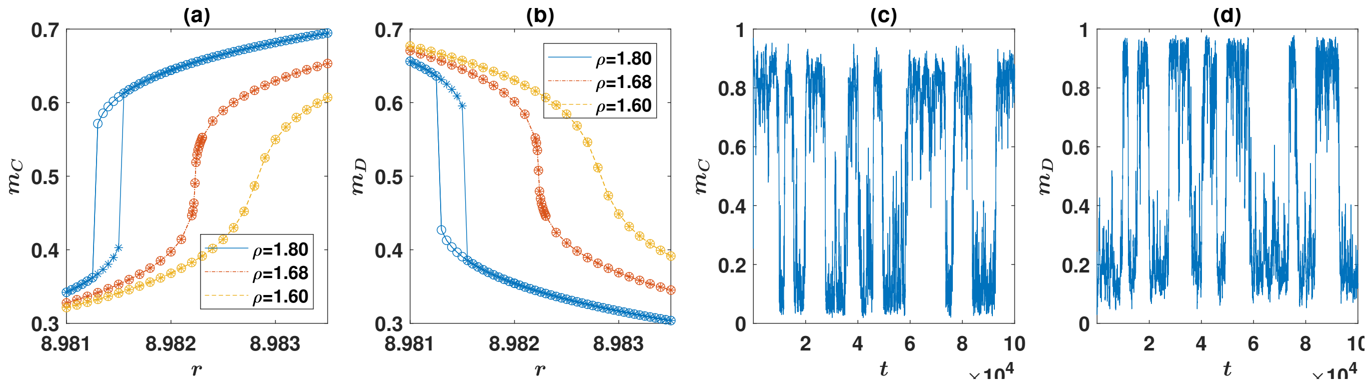

FIG. S.3. The nature of the $C-D$ transition in a well-mixed population. (a) and (b): the equilibrium frequency of non-punishing cooperators, m_C, (a), and the equilibrium frequency of non-punishing defectors m_D, (b), as a function of r for three different values of ρ. Two different initial conditions are used: one in which all the individuals are non-punishing cooperators (circles) and one in which all the individuals are non-punishing defectors (stars). While for large ρ, close to the $C-D$ transition, the system shows bi-stability indicating a discontinuous transition, there is a cross-over from the D phase to the C phase for small ρ. In between, the transition becomes a continuous transition at a critical value of ρ. (c) and (d): The frequency of non-punishing cooperators, m_C, (a), and the frequency of non-punishing defectors m_D, (b), resulted from a simulation in a population of size $N = 10000$, as a function of time. The dynamics show intermittency between the two phases. In all the cases, $g = 9$, $\nu = 0.001$, $c = c' = 1$, and $\alpha = 0.5$. In (c) and (d), $\rho = 2$, and $r = 9$.

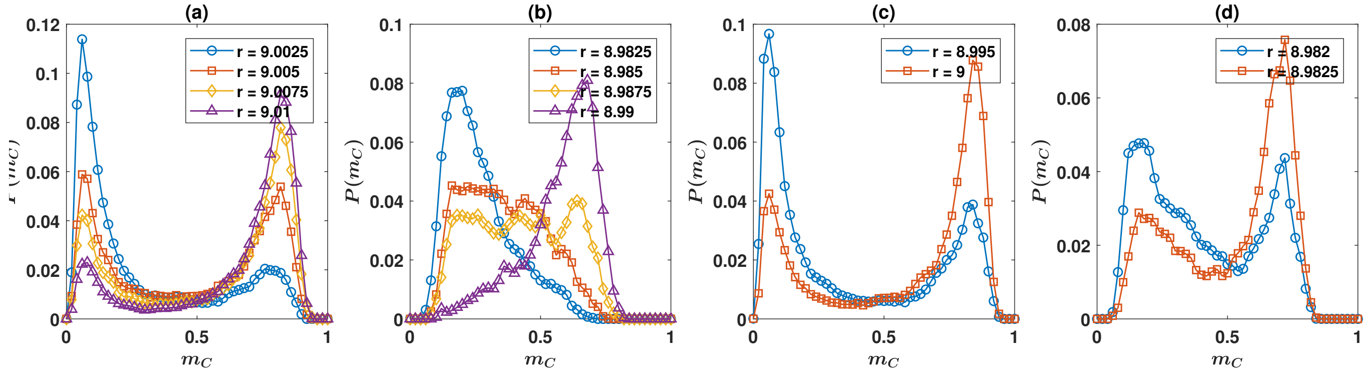

FIG. S.4. The nature of the $C-D$ transition in a well-mixed population. The distribution of m_C for $\rho = 1.25$, (a) and (b), and for $\rho = 2$, (c) and (d), resulting from a simulation. In (a) and (c) the population size is $N = 10000$ and in (b) and (d) the population size is $N = 80000$. While in a small population both distributions show bi-modality, in larger populations, the two cases are different. For small ρ, (b), the distribution becomes uni-modal, and for large ρ, (d), the distribution remains bi-modal in larger populations, as well. This shows the $C-D$ transition possesses strong finite size effects. Here, $g = 9$, $\nu = 0.001$, $c = c' = 1$, and $\alpha = 0.5$. The distribution are derived from a simulation of the system run for $T = 150000$ time steps, after discarding the first 1000 time steps.

cooperators is one, and all the other strategies have a frequency of zeros in the population. The equilibrium state of this initial condition is indicated in the figure by circles. As the second initial condition, we solve the replicator dynamics starting from an initial condition in which the initial frequency non-punishing defectors equals one, and all the frequency of all the other strategies is equal to zero. This is indicated by stars in the figure.

As can be seen, for large ρ ($\rho = 1.8$), close to the $C-D$ transition, the system shows bistability. This indicates that the $C-D$ transition is discontinuous in this region. However, we note that, contrary to the other phases, the coexistence region of the C and the D phases spans a small region close to this transition. On the other hand, for small values of ρ ($\rho = 1.6$), as r increases, the system shows a cross over from the D phase to the C phase without passing any singularity. In between, the transition becomes a singular transition, at a critical value of ρ and r.

The results of simulations appear to show a similar phenomenology, as expected. An example of time series of the system close to the $C-D$ transition, resulting from a simulation in a population of size $N = 10000$ is presented in Fig. (S.3.c) and Fig. (S.3.d). Here, we plot the frequency of non-punishing cooperators m_C, and the frequency of non-punishing defectors m_D, as a function of time. Here, we have fixed $g = 9$, $\rho = 2$, and $\nu = 0.001$, and have chosen $r = 9$, which lies close to the $C-D$ transition. As can be seen, in a finite population, the system shows intermittency

between the two phase, which suggest a discontinuous transition. However, simulations in small populations even for small values of ρ show a similar intermittency. This can be seen in Fig. (S.4.a), where the distribution of m_C, resulted from a simulation in a population of size $N = 10000$ is plotted. Here $\rho = 1.25$. In Fig. (S.4.c), the same distribution for a population of size $N = 10000$ for $\rho = 2$ is plotted. As can be seen in both cases, the distribution is bi-modal, which should be the case in a discontinuous transition. However, going to larger system sizes, the situation is different for small and large ρ. This can be seen in Fig. (S.4.b) and Fig. (S.4.d), where the distribution of m_C for respectively, $\rho = 1.25$ and $\rho = 2$, for a population of size $N = 80000$ is plotted. As can be seen, going to a large population, while the distribution of m_C remains bi-modal for large ρ, it becomes a uni-modal distribution for small ρ, as expected from the solutions of the replicator dynamics. We conclude that, close to the $C - D$ transition, the system shows strong finite size effect such the the $C - D$ transition appears to be discontinuous even for small ρ, in a small size system.

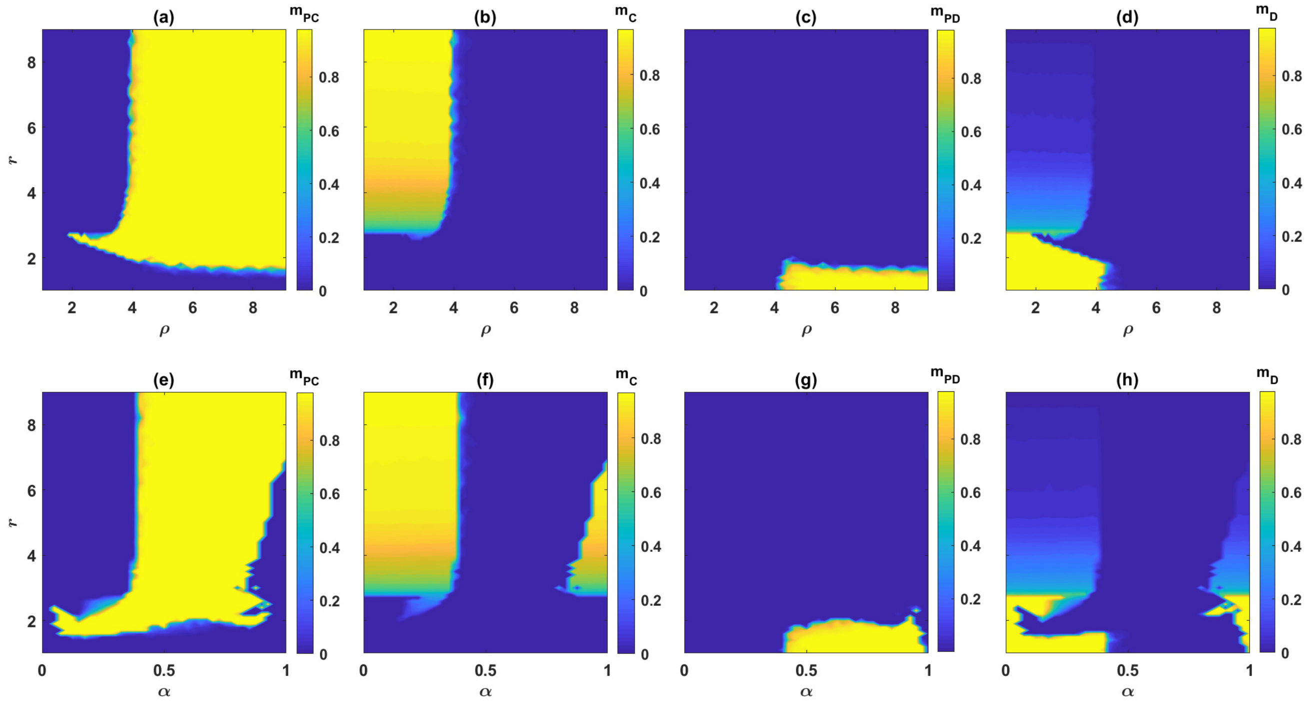

FIG. S.5. Contour plot of the frequency of different strategies in a structured population, in the $\rho - r$ plane ((a) to (d)), and in the $\alpha - r$ plane ((e) to (h)). Here, a population of size $N = 40000$ individuals residing on a 200×200 square lattice with Moore connectivity and periodic boundaries is considered. The simulations are started from a random initial condition in which the strategies of the individuals are assigned at random. Here, $\nu = 10^{-3}$, $c = c' = 1$, in (a) to (d) $\alpha = 0.5$ and in (e) to (h), $\rho = 5$. The simulations are run for $T = 1000$ time steps, and an average over the last 100 time steps is taken.

S. 4. ANALYSIS OF THE MODEL: STRUCTURED POPULATIONS

S. 4.1. The frequency of different strategies

The densities of different strategies in the case of the structured population are presented in Fig. (S.5). In Fig. (S.5.a) to Fig. (S.5.d), we present the densities of different strategies in the $r - \rho$ plane, and in Fig. (S.5.e) to Fig. (S.5.h), we present the densities of different strategies in the $r - \alpha$ plane. Fig. (S.5.a) and Fig. (S.5.e) present m_{PC}, Fig. (S.5.b) and Fig. (S.5.f) present m_C, Fig. (S.5.c) and Fig. (S.5.g) present m_{PD}, and Fig. (S.5.d) and Fig. (S.5.h) show m_D. Here, simulations are performed on a population of size $N = 40000$ individuals residing on a 200×200 first nearest neighbor two dimensional square lattice, with Moore connectivity and periodic boundaries. In Fig. (S.5.a) to Fig. (S.5.d), we have set $g = 9$, $\nu = 10^{-3}$, and $\alpha = 0.5$, and in Fig. (S.5.e) to Fig. (S.5.h), we have set $g = 9$, $\nu = 10^{-3}$, and $\rho = 5$. The phase diagrams presented in the main text are derived from these results by locating the transition lines and different phases.

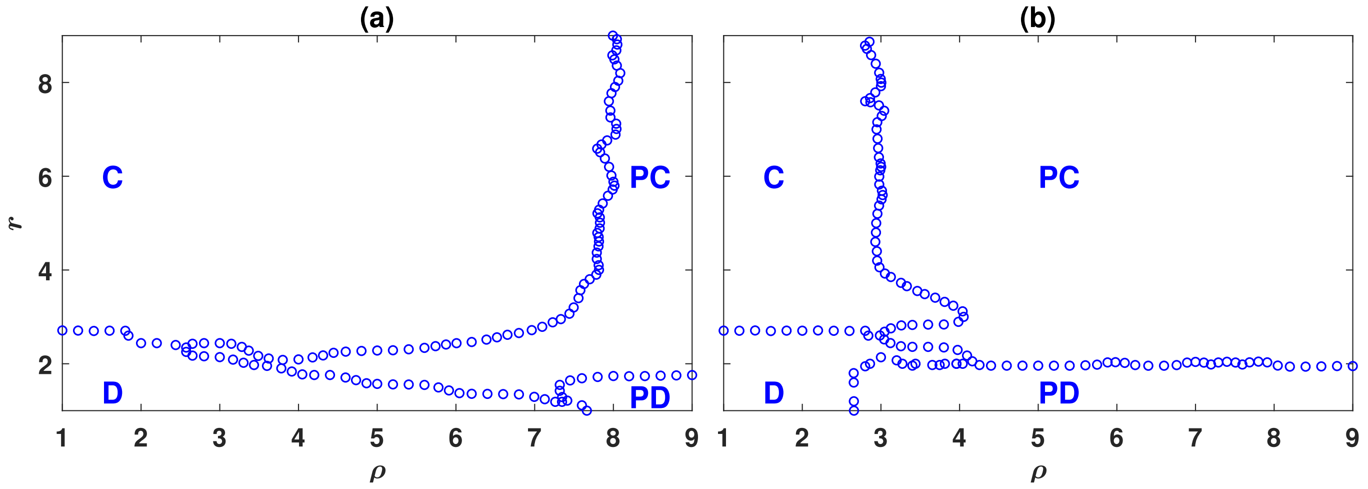

FIG. S.6. Evolution of social punishment is facilitated near a phase transition. The phase diagram of the model for a structured population for two different values of α. In (a), $\alpha = 0.25$ and in (b), $\alpha = 0.75$. For both values of α, close to the $C - D$ phase transition, the transition to the social punishment requires smaller punishment enhancement factors. The phase diagram is derived by running simulations in a population of size 40000, residing on a 200×200 first nearest neighbor lattice with Moore connectivity and periodic boundaries. Here, $g = 9$, $\nu = 0.001$, and $c = c' = 1$.

We begin our analysis, by investigating the behavior of the system in the $r - \rho$ plane. As seen in the phase diagram of the model presented in the main text, the system can settle in four different phases, in each of which one of the four possible strategies dominates. For small ρ, such that the return to the investment in the punishment pool is small, non-punishing strategies evolve. Here, for small r the system settles in the D phase, in which non-punishing defectors dominate. By increasing r, for a small ρ, a phase transition to a phase in which non-punishing cooperators survive (which we call the C phase) occurs. We note that, for a mixed population, this transition occurs for a value of r close to $g = 9$, where, investment to the public pool yields a positive payoff to an individual, and thus, it is no longer a dilemma. On the other hand, as can be seen in the figure, for a structured population this transition occurs for a much smaller value of r (smaller than 3), where investment to the public pool results in a negative payoff for the investor, and thus, constitutes a social dilemma. This shift in the transition is due to the facilitating effect of network structure for the evolution of cooperation. In addition, comparison with a similar model, in which only non-punishing strategies exist, shows the $C - D$ transition shifts to smaller values compared to such a model, with the same size and network structure. This is due to the positive effect of the existence of the punishing strategies for the evolution of cooperation.

As the value of ρ increases, punishing strategies evolve. Here, for very small r, the PD phase, in which punishing defectors dominate the population occurs. As r increases, for fixed large enough ρ, a transition to the PC phase in which punishing cooperators dominate the population occurs. We note that, for a fixed large enough ρ, the value of r where the transition from the anticosial punishment to the social punishment evolves is smaller than the value or r where the transition from the D phase to the C phase occurs. Furthermore, by increasing ρ, the $PD - PC$ transition line shifts to smaller values of r. This interesting observation results from the beneficial effect of punishment for the evolution of cooperation, such that the larger ρ and the more effective the punishment, the easier cooperative strategies can evolve.

Finally, we note that the evolution of social punishment is facilitated close to the $C - D$ transition. This can be seen by noting that the value of ρ for which the system settles in the PC phase significantly decreases close to the $C - D$ transition. This interesting observation shows that being close to a physical phase transition, facilitates the evolution of social punishment.

We turn to the densities of different strategies in the $r - \alpha$ plane, presented in Fig. (S.5.e) to Fig. (S.5.h). As can be seen in the figure, for small values of α, such that the punishment of second-order free-riding is not strong enough, punishing strategies do not evolve. Instead, here for small values of r, non-punishing defectors dominate, and as r increases, the system shows a transition to a phase where non-punishing cooperators can survive. Similarly, for small enough values of r, and for too large values of α, such that the punishment of defectors by social punishers (or the punishment of cooperators by antisocial punishers) is too weak, punishing strategies do not evolve and the system settles in either the D phase (for smaller r) or the C phase (for larger r). However, when r is very large (larger than ~ 6), social punishment can evolve, even when $\alpha = 1$. That is when social punishers do not punish defectors. This

was not the case in the case of a mixed population and results from strong network reciprocity for very large values of r. For values of α, in between these two extremes, punishing strategies evolve. Here, for small values of r antisocial punishment evolves, and as r increases, the system shows a phase transition to a phase where social punishment evolves.

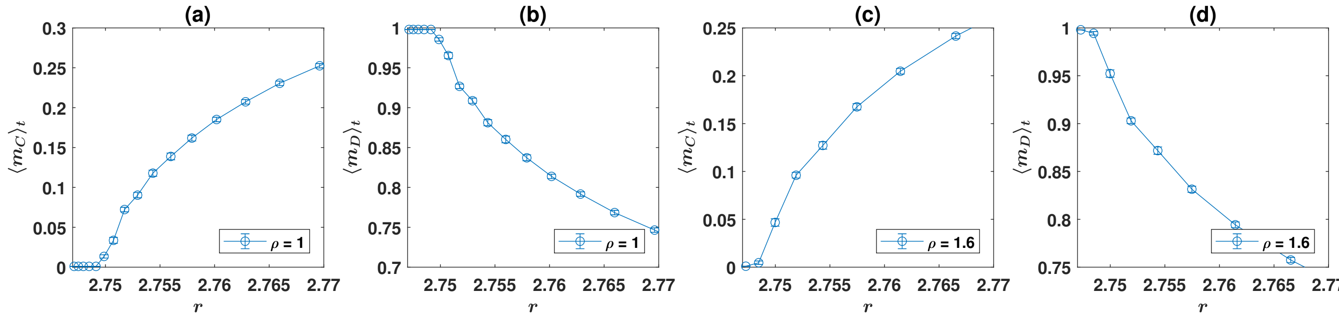

FIG. S.7. The nature of the $C-D$ transition in a structured population. (a) to (d): The time average densities of non-punishing cooperators (a and c) and non-punishing defectors (b and d) as a function of r, close to the $C-D$ transition for two different values of ρ as indicated in the figure. For a fixed small value of ρ, for r smaller than a critical value, non-punishing defectors dominate the population. As r increases beyond a critical value, non-punishing cooperators can survive. The transition between the two phases shows no discontinuity and appears continuously, for all values of ρ. Here, the simulations are performed in a population of size $N = 640000$, living on a 800×800 square lattice, with periodic boundaries and Moore connectivity. The mutation rate is set equal to $\nu = 0.001$. The simulations are run for $T = 50000$ time steps and the time averages are taken over the last 20000 time steps of the simulations.

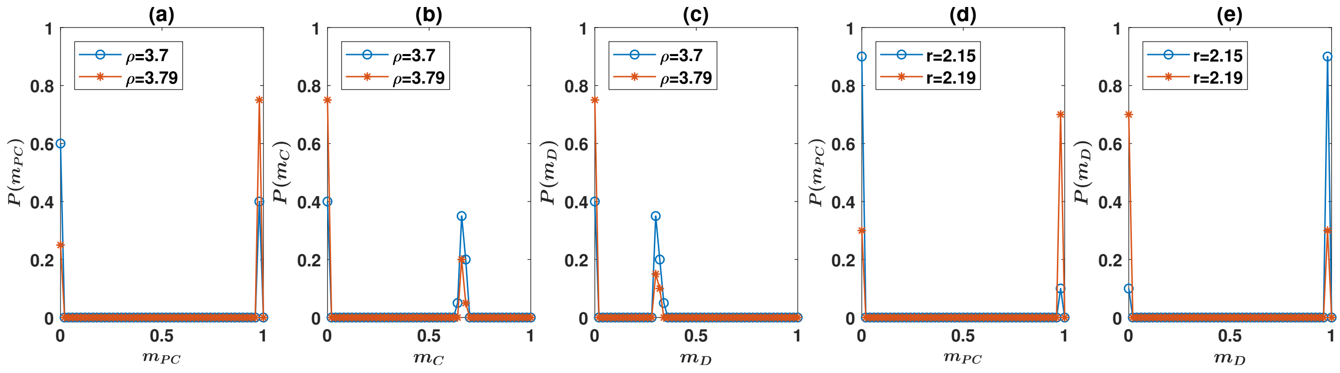

FIG. S.8. The $C-PC$ and $D-PC$ transitions are discontinuous. (a) to (c): The $C-PC$ transition shows bi-stability and is discontinuous. The distribution of m_{PC} (a), m_C (b), and m_D (c), for $r = 4$ and two different values of ρ chosen close to the $C-PC$ transition. All the distributions are bimodal, which shows the $C-PC$ transition is discontinuous. The peak corresponding to a small value of m_{PC}, and large values of m_C and m_D corresponds to the C phase, and the peak corresponding to a value of m_{PC} close to 1, and m_C and m_D close to 0, corresponds to the PC phase. By increasing ρ, the peak corresponding to the C phase decreases, while that corresponding to the PC phase increases. (d) and (e): The distribution of m_{PC} (d) and m_D (e), for $\rho = 3.2$ and two different values of r, chosen close to the $D-PC$ transition. The distributions are bimodal, which shows the $D-PC$ transition is discontinuous. The peak with m_{PC} close to 0 and m_D close to 1 corresponds to the D phase, while that with m_{PC} close to 1 and m_D close to 0 corresponds to the PC phase. By increasing r the peak corresponding to the D phase decreases, while that corresponding to the PC phase increases. This shows the $D-PC$ transition is discontinuous. In all the panels, the network is a two dimensional 100×100 square lattice with Moore connectivity and periodic boundaries, and $\nu = 0.005$. The distributions are derived from the final state of a sample of $R = 20$ simulations, starting from random initial conditions. In (a) to (c), the simulations are run for $T = 6000$ time steps, and in (d) and (e), the simulations are run for $T = 10000$ time steps.

S. 4.2. The evolution of social punishment is facilitated close to a phase transition

We have seen that close to the $C-D$ phase transition, the evolution of social punishment is facilitated, as it happens for smaller values of the punishment enhancement factor, ρ. In this section we argue that this result is robust

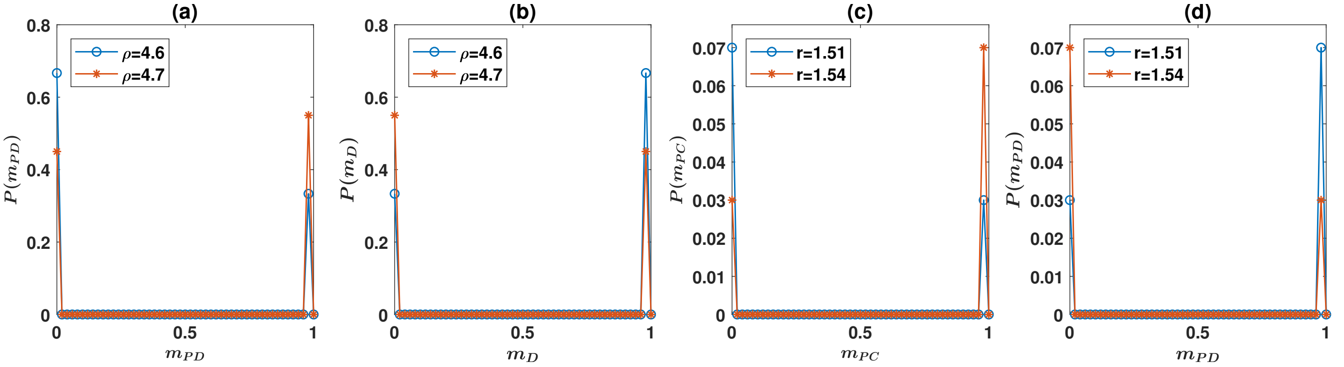

FIG. S.9. The $D-PD$ and $PD-PC$ transitions are discontinuous. (a) and (b): The distribution of m_{PD} (a), and m_D (b), for $r = 1.2$ and two different values of ρ chosen close to the $D-PD$ transition. As can be seen, these distributions are bimodal. The peak corresponding to a value of m_{PD} close to 0, and m_D close to 1, corresponds to the D phase, and the peak corresponding to a value of m_{PD} close to 1, and m_D close to 0, corresponds to the PD phase. By increasing ρ, the peak corresponding to the D phase decreases, while that corresponding to the PD phase increases. This shows the $D-PD$ transition is discontinuous. (c) and (d): The distribution of m_{PC} (c), and m_{PD} (d), for $\rho = 5$ and two different values of r, chosen close to the $PD-PC$ transition. The distributions are bimodal, which shows the $PD-PC$ transition is discontinuous. The peak with m_{PC} close to 0 and m_{PD} close to 1 corresponds to the PD phase, while that with m_{PC} close to 1 and m_{PD} close to 0 corresponds to the PC phase. By increasing r the peak corresponding to the PD phase decreases, while that corresponding to the PC phase increases. In all the panels, the network is a two dimensional 100 lattice with periodic boundaries and Moore connectivity (in (a) and (b)), or Von Neuman connectivity (in (c) and (d)). In (a) and (b) $\nu = 0.005$, and in (c) and (d), $\nu = 0.001$. The distributions are derived from the final state of a sample of $R = 10$ simulations, starting from random initial conditions. In (a) and (b), the simulations for $\rho = 4.6$ (blue curve marked with circle) is run for $T = 50000$ time steps, and that for $\rho = 4.7$ (red curve marked with stars) is run for $T = 75000$ time steps, and in (d) and (e), the simulations are run for $T = 10000$ time steps. In (c) and (d), the simulations are run for $T = 30000$ time steps.

and occurs for different parameter values. To do this, we run simulations for two different values of α to drive the phase diagram of the system. The results are presented in Fig. (S.6). In Fig. (S.6.a), $\alpha = 0.25$ and in Fig. (S.6.b), $\alpha = 0.75$. Here, the phase diagram is derived by running simulations in a population of size 40000, residing on a 200×200 first nearest neighbor lattice with Moore connectivity and periodic boundaries. Here, $g = 9$, and $\nu = 0.001$. As can be seen, for both values of α, close to the $C-D$ phase transition, the transition to the social punishment requires smaller punishment enhancement factors. The shift is more significant for smaller α, where away from the phase transition, a relatively large value of ρ is necessary for the evolution of both social and antisocial punishment. In contrast, the transition to the social punishment phase shifts to significantly smaller values of ρ close to the $C-D$ phase transition.

For larger values of α, as in Fig. (S.6.b), the evolution of social punishment is facilitated and it can happen for smaller values of ρ. In addition, for larger enhancement factors, r, the transition to the social punishment is easier as it requires smaller punishment enhancement factors, ρ. However, close to the $C-D$ transition, this transition shifts to smaller values of ρ. This suggests that being close to a phase transition facilitates the evolution of social punishment in this case, as well.

S. 4.3. The nature of the phase transitions

In this section, we show that all the transitions which involve punishing strategies, that is $C-PC$, $D-PC$, $D-PD$, and $PD-PC$ show bi-stability and are discontinuous. On the other hand, the $C-D$ transition appears to be a continuous transition.

We begin by the $C-D$ transition, which has a different nature from the other transitions. As mentioned before, for each value of ρ, small enough, as r increases a phase transition from the D phase, in which non-punishing defectors dominate the population, to the C phase, in which non-punishing cooperators dominate occurs. We call this transition the $C-D$ transition. To study this transition, we perform simulations in a population of size $N = 640000$, living on a first nearest neighbor two dimensional 800×800 square lattice, with periodic boundaries and Moore connectivity. The results of simulations for two different values of ρ are presented in Fig. (S.7). Here, the mutation rate is set $\nu = 0.001$, and the densities of non-punishing cooperators and non-punishing defectors are plotted.

As can be seen, for small r non-punishing defectors dominate the population, with a frequency close to 1. In this phase, in addition to non-punishing defectors, a small fraction of other strategies are maintained in the population,

due to mutations. As r increases beyond a pseudo-critical point, non-punishing cooperators start to survive. As shown below, they do so, by forming compact domains in which the benefit of cooperation is reaped by the fellow cooperators. Close to the pseudo-critical point, by increasing r, the frequency of non-punishing cooperators rapidly increases, and that of the non-punishing defectors rapidly decreases. The change in the densities of all the strategies occurs continuously, and no discontinuity in this transition is observed. This suggests that the $D-C$ transition is a continuous transition.

We begin the study of the discontinuous transitions by the $C-PC$ transition. To this goal, in Fig. (S.8.a) to Fig. (S.8.c), we plot the distribution of, respectively, m_{PC}, m_C, and m_D. Here, a population of $N = 10000$ individuals living on a first nearest neighbor two dimensional 100×100 lattice with Moore connectivity and periodic boundaries is considered. The mutation rate, is set equal to $\nu = 0.005$. The distributions are derived from the final state of a sample of $R = 20$ simulations, after $T = 6000$ time steps of evolution, starting from random initial conditions. Here, we have set $r = 4$ and the distributions are plotted for two different values of ρ chosen close to the $C-PC$ transition. As can be seen in the figure, the distributions are bimodal and have two peaks. This shows that the $C-PC$ transition is discontinuous. The peak corresponding to a small value of m_{PC}, and large values of m_C and m_D corresponds to the C phase, in which cooperators survive and can coexist with defectors. On the other hand, the peak corresponding to a value of m_{PC} close to 1, and m_C and m_D close to 0, corresponds to the PC phase, in which punishing cooperators dominate the population. By increasing ρ, the peak corresponding to the C phase decreases, while that corresponding to the PC phase increases. This phenomenology is characteristic of a discontinuous transition.

The nature of the $D-PC$ transition is investigated in Fig. (S.8.d), where the distribution of m_{PC} is plotted, and Fig. (S.8.e), where the distribution of m_D is plotted. Here, a population of $N = 10000$ individuals living on a first nearest neighbor two dimensional 100×100 lattice with Moore connectivity and periodic boundaries is considered. The mutation rate is set equal to $\nu = 0.005$. The distributions are derived from the final state of a sample of $R = 20$ simulations, after $T = 10000$ time steps of evolution starting from random initial conditions. Here, we have set $\rho = 3.2$ and the distributions are plotted for two different values of r, as indicated in the figures. These values are chosen such that the system is posed close to the $D-PC$ transition. As can be seen, the distributions have two peaks and are bimodal. The peak with m_{PC} close to 0 and m_D close to 1 corresponds to the D phase, while that with m_{PC} close to 1 and m_D close to 0 corresponds to the PC phase. By increasing r the peak corresponding to the D phase decreases, while that corresponding to the PC phase increases. This phenomenology is characteristic of a discontinuous phase transition and shows that the $D-PC$ transition is discontinuous.

The nature of the $D-PD$ transition is investigated in Fig. (S.9.a) and Fig. (S.9.b). In Fig. (S.9.a), the distribution of m_{PD} is plotted, and in Fig. (S.9.b), the distribution of m_D is plotted. Here, a population of $N = 10000$ individuals living on a first nearest neighbor two dimensional 100×100 lattice with Moore connectivity and periodic boundaries is considered. The mutation rate, is set equal to $\nu = 0.005$. The distributions are derived from the final state of a sample of $R = 20$ simulations, starting from random initial conditions. Here, we have set $r = 1.2$ and the distributions are plotted for two different values of ρ, as indicated in the figures, chosen such that the system is posed close to the $D-PD$ transition. The simulations for $\rho = 4.6$ (blue curve marked with circle) is run for $T = 50000$ time steps, and that for $\rho = 4.7$ (red curve marked with stars) is run for $T = 75000$ time steps. As can be seen, the distributions have two peaks and are bimodal. The peak with m_{PD} close to 0 and m_D close to 1 corresponds to the D phase, while that with m_{PD} close to 1 and m_D close to 0 corresponds to the PD phase. By increasing ρ, the peak corresponding to the D phase decreases, while that corresponding to the PD phase increases. This phenomenology is characteristic of a discontinuous phase transition and shows that the $D-PD$ transition is discontinuous.

Finally, the nature of the $PD-PC$ transition is investigated in Fig. (S.9.c) and Fig. (S.9.d). In Fig. (S.9.c), the distribution of m_{PC} is plotted, and in Fig. (S.9.d), the distribution of m_{PD} is plotted. Here, a population of $N = 10000$ individuals living on a first nearest neighbor two dimensional 100×100 lattice with von Neumann connectivity (that is each site is connected to four sites, to its north, south, east, and west) and periodic boundaries is considered. The mutation rate, is set equal to $\nu = 0.001$. The distributions are derived from the final state of a sample of $R = 10$ simulations, starting from random initial conditions. The simulations are run for $T = 30000$ time steps. Here, we have set $\rho = 5$ and the distributions are plotted for two different values of r, as indicated in the figures, chosen such that the system is posed close to the $PD-PC$ transition. As can be seen in the figure, the distributions are bimodal, which shows the $PD-PC$ transition is discontinuous. The peak with m_{PC} close to 0 and m_{PD} close to 1 corresponds to the PD phase, in which punishing defectors dominate. On the other hand, the peak with m_{PC} close to 1 and m_{PD} close to 0 corresponds to the PC phase, in which punishing cooperators dominate the population. By increasing r the peak corresponding to the PD phase decreases, while that corresponding to the PC phase increases. This shows the $PD-PC$ transition is discontinuous.

We note that, contrary to the other cases, where we have used a lattice with Moore connectivity, here, a different connectivity, von Neumann connectivity is used. The reason is that, as it will be explained shortly, the dynamics of the system close to the discontinuous transitions is driven by slow growth of domains of individuals with the same strategy, in a sea of individuals with another, competing strategy. On a network with Moore connectivity, these domains are

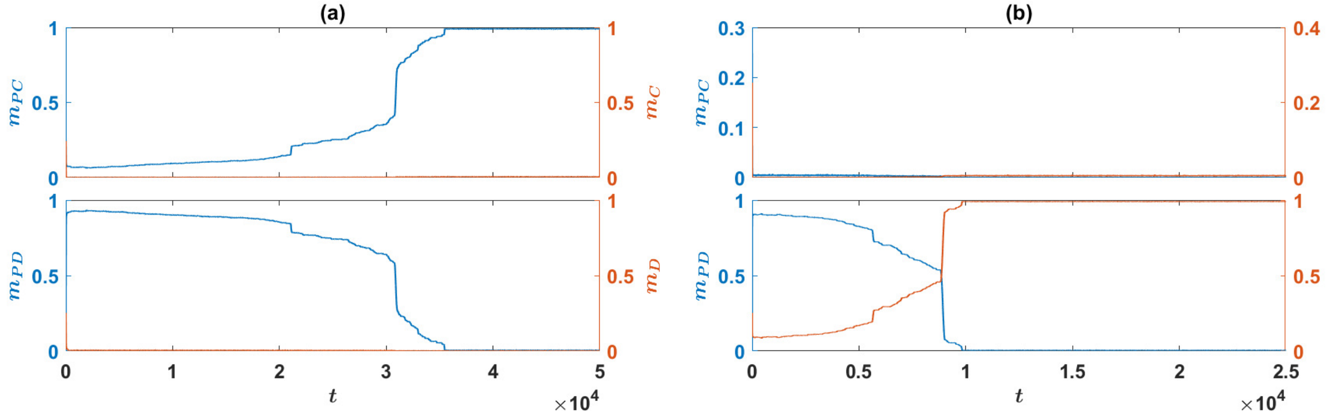

FIG. S.10. Time evolution of the frequency of different strategies close to the $D - PC$ transition. In (a), $r = 1.72$ and $\rho = 5$, and in (b), $r = 1.2$ and $\rho = 4.65$. In both cases $c = c' = 1$, $\nu = 0.005$, and the population resides on a 100×100 square lattice with Moore connectivity and periodic boundaries. In both cases, the time evolution of the system shows two different time scales: a slow growth, intermittent with short periods of fast growth. This time evolution is characteristic of the system close to all the discontinuous transitions. The slow growth corresponds to slow growth of homogeneous blocks along their vertical and horizontal boundaries, and the fast growth sets in when different blocks merge, which results in non- horizontal and non-vertical boundaries, along which the growth of a homogeneous block happens in a much faster speed.

rectangular domains, and the growth of the domains proceeds along their horizontal and vertical boundaries. As close to the transition, such a growth can be very slow, the time needed for the system to reaches equilibrium can be excessively large. This is the case for all the discontinuous transitions, but particularly, prominent for the case of the $PC - PD$ transition. As the nature of the transition is the same for both Moore and von Neumann connectivity, we have changed the network connectivity to von Neumann connectivity, where the equilibration time of the system is shorter, and thus, it is more convenient to study.

S. 4.4. Growth process

We end this section, by taking a deeper look into the growth process of different strategies close to the discontinuous transitions. To this goal, in Fig. (S.10), we present the time evolution of the frequency of different strategies, close to the $D - PC$ transition. Here, $\nu = 0.005$, and the population resides on a first nearest neighbor 100×100 two dimensional lattice with Moore connectivity and periodic boundaries. In (Fig. (S.10.a), $r = 1.72$ and $\rho = 5$, These parameters are chosen such that the system is close to the $PC - PD$ transition, but in the PC phase. In Fig. (S.10.b), $r = 1.2$ and $\rho = 4.65$. These parameters are chosen such that the system is close to the $D - PD$ transition, but in the D phase. As can be seen, in both cases the time evolution of the system shows two different time scales: a slow growth intermittent with short periods of fast growth. This gives the resulting time series a staircase-like appearance. To see why this is the case, in Fig. (S.11), Fig. (S.12), and Fig. (S.13), we present snapshots of the time evolution of the system close to different phase transition. In Fig. (S.11.e) to Fig. (S.11.h), snapshots of the time evolution close to the $C - PC$ phase transition are represented, in Fig. (S.12.a) to Fig. (S.12.d), and also in Fig. (S.12.e) to Fig. (S.12.h), snapshots of the time evolution of two simulations for different parameter values, both chosen close to the $D - PC$ phase transition are presented, and in Fig. (S.13.a) to Fig. (S.13.d), snapshots of the time evolution of the system close to the $D - PD$ phase transition are presented, and finally, in Fig. (S.13.e) to Fig. (S.13.h), snapshots of the time evolution of the system close to the $PD - PC$ phase transition are presented. In all the cases, the population lives on a first nearest neighbor two dimensional 300×300 lattice with periodic boundaries and Moore connectivity. See the Figures for more detail.

As can be seen in the figures, in all the cases, close to the discontinuous transition, the dynamics involves (at least) two stages. Starting from a random initial condition, at the first stage of the evolution, one of the strategies rapidly grows and forms a sea of homogeneous strategies. Usually, one (in some cases more) of the strategies survive by forming compact rectangular blocks. The second stage of the evolution begins by the slow growth of these rectangular domains along their horizontal and vertical boundaries. The long periods of semi-stasis observed in Fig. (S.10),

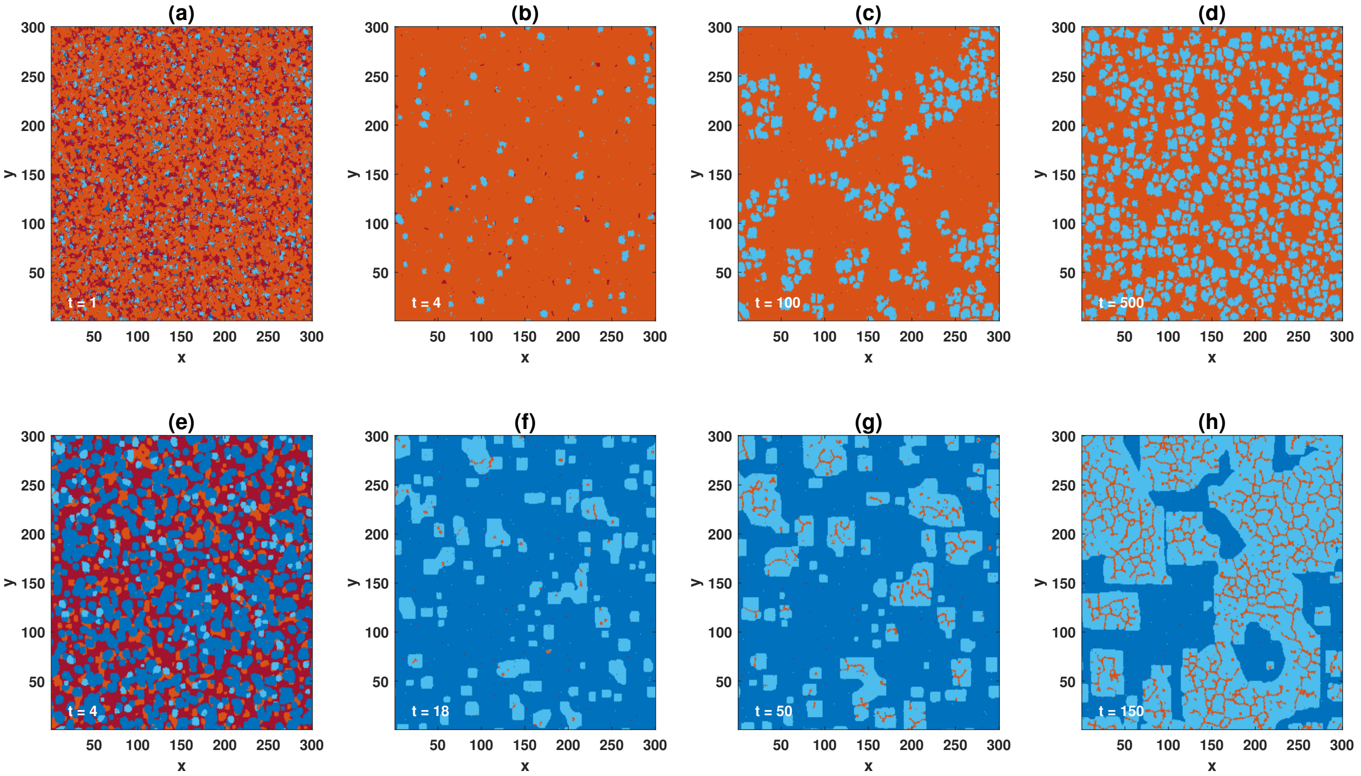

FIG. S.11. Time evolution of the system, close to the $C-D$ phase transition ((a) to (d)), and close to the $C-CP$ phase transition ((e) to (h)). Different strategies are indicated by different colors. Light blue shows non-punishing cooperators, dark blue shows punishing cooperators, light red shows non-punishing defectors, and dark red shows punishing defectors. (a) to (d): Starting from a random initial condition, close to the $C-D$ transition, defectors rapidly increase in frequency and dominate the population. While all the other strategies go to extinction, non-punishing cooperators can survive by forming compact domains in which the benefit of cooperation is reaped by fellow cooperators. The dynamics is governed by formation, division, and collapse of cooperator blocks in the sea of defectors. (e) to (h): Close to the $C-PC$ transition, starting from a random initial condition, punishing cooperators rapidly expand by driving all the other strategies into extinction. Only non-punishing cooperators can survive by forming small rectangular domains. These blocks slowly increase in the see of punishing cooperators, until they expand the whole population. While defectors can not survive in the sea of punishing cooperators, they survive by forming narrow bands in the non-punishing cooperators' communities. Here, a population of $N = 90000$ individuals lives on a two dimensional 300×300 lattice with periodic boundaries and Moore connectivity. In (a) to (d) $r = 2.8$ and $\rho = 1.4$, and in (e) to (h), $r = 4$ and $\rho = 3.47$. In all the cases $\nu = 10^{-3}$, and $c = c' = 1$.

results from such long periods of slow growth. However, at some time instances, two or more rectangular blocks meet, resulting in a non-rectangular island of individuals with the same growing strategy, in a sea of individuals with the same, but different strategy. Interestingly, the growth of such non-rectangular shapes posses a much faster time scale, resulting in the short periods of rapid growth observed in Fig. (S.10).

Finally, for some parameter values, a rock-paper-scissor like dynamics is observed. An example is presented in Fig. (S.12.a) to Fig. (S.12.d), and in the Supplementary Video 3. In this case, starting from a random initial condition, punishing defectors rapidly grow and form a sea of punishing defectors, in which other strategies can only survive by forming small islands of homogeneous strategies. While punishing defectors can drive blocks of non-punishing and punishing cooperators into extinction, they are dominated by growing blocks of non-punishing defectors. These later blocks start invading the sea of punishing defectors until driving them to extinction. Although punishing defectors could have beaten punishing cooperators, this is not the case for non-punishing defectors. Once non-punishing defectors wash out punishing defectors, blocks of punishing cooperators surrounded by non-punishing defectors can grow until driving non-punishing defectors into extinction.

Another example of such rock-paper-scissor like dynamics occurs close to the $C-PC$ transition, Fig. (S.11.e) to Fig. (S.11.h) and Supplementary video 1. Here, punishing cooperators pave the way for the invasion of non-punishing cooperators, by driving defectors into extinction. When immune from defectors, blocks of non-punishing cooperators can outperform and grow in the sea of punishing cooperators. Non-punishing defectors, while in disadvantage in the presence of punishing cooperators, reappear in the system, once punishing cooperators get eliminated by non-

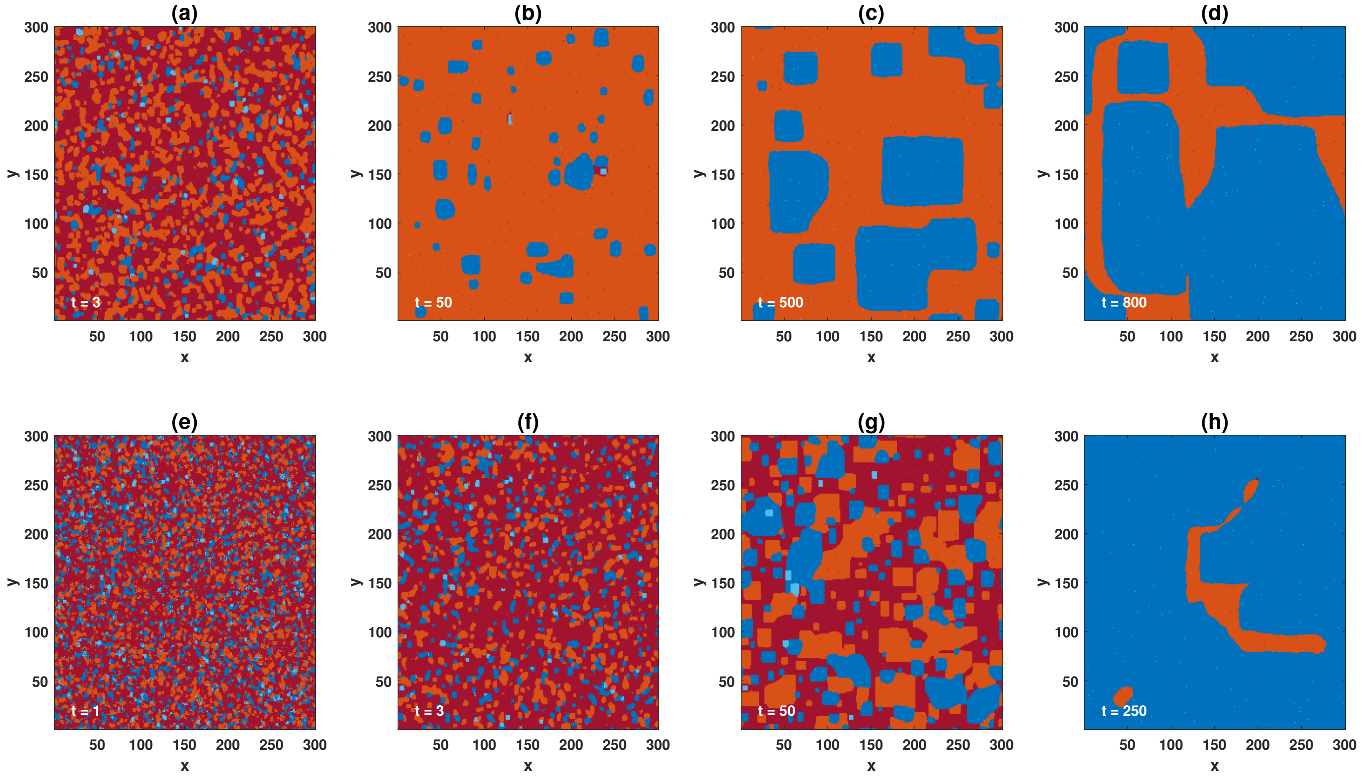

FIG. S.12. Time evolution of the system, close to the $D - PC$ phase transition. In (a) to (d), $r = 2.2$ and $\rho = 3.2$, and in (e) to (h), $r = 2.1$ and $\rho = 3.8$. Different strategies are indicated by different colors. Light blue shows non-punishing cooperators, dark blue shows punishing cooperators, light red shows non-punishing defectors, and dark red shows punishing defectors. Starting from a random initial condition, close to the $C - PC$ transition, one of the strategies (non-punishing defectors in (a) to (d), and punishing defectors in (e) to (h)) rapidly increase in frequency and form a sea of homogeneous strategies. Other strategies can only survive by forming compact domains. The second stage of the evolution is governed by slow growth of domains of punishing cooperators along their boundaries. In both cases, a population of $N = 90000$ individuals lives on a two dimensional 300×300 lattice with periodic boundaries and Moore connectivity. Here, $\nu = 10^{-3}$, and $c = c' = 1$.

punishing cooperators. We note that, this rock-paper-scissor like dynamic can facilitate the evolution of cooperation, due to elimination of defectors by punishing cooperators, close to the $C - D$ transition. Consequently, in our model, the $C - D$ transition shifts to smaller values of enhancement factors, r, compared to a system with the same network structure and size in a model where punishing strategies are absent.

S. 5. SUPPLEMENTARY VIDEOS

In the Supplementary Videos (SV), the time evolution of the system, for a population of size $N = 90000$ residing on a 2-dimensional 300×300 first nearest neighbor square lattice with Moore connectivity and periodic boundaries is presented. In the videos, we fix $\nu = 10^{-3}$ and $\alpha = 0.5$, and change the values of r and ρ, such that the system is posed close to different phase transitions.

In SV.1 we have chosen $r = 4$ and $\rho = 3.47$. With these values, the system is in the C phase, close to the $C - PC$ phase transition. As can be seen in the video, starting from a random initial condition, the defective strategies, D and PD, are eliminated rapidly by punishing cooperators (PC). However, small rectangular-shape islands of non-punishing cooperators (C) are formed in the sea of punishing cooperators. As here, the parameters of the model are chosen such that the system is in the C phase, non-punishing cooperators are in advantage with respect to punishing cooperators. Consequently, the small islands of non-punishing cooperators start to grow along the horizontal and vertical boundaries, until they invade the whole population. Non-punishing defectors, although unable to survive in the sea of punishing cooperators, can survive in the islands of non-punishing cooperators by forming narrow bands. Consequently, the frequency of non-punishing defectors increases as well, with increasing the frequency of non-punishing cooperators.

In the SV.2, we have set $r = 2.8$, and $\rho = 1.4$. With these values, the system is close to the $C - D$ transition, in the

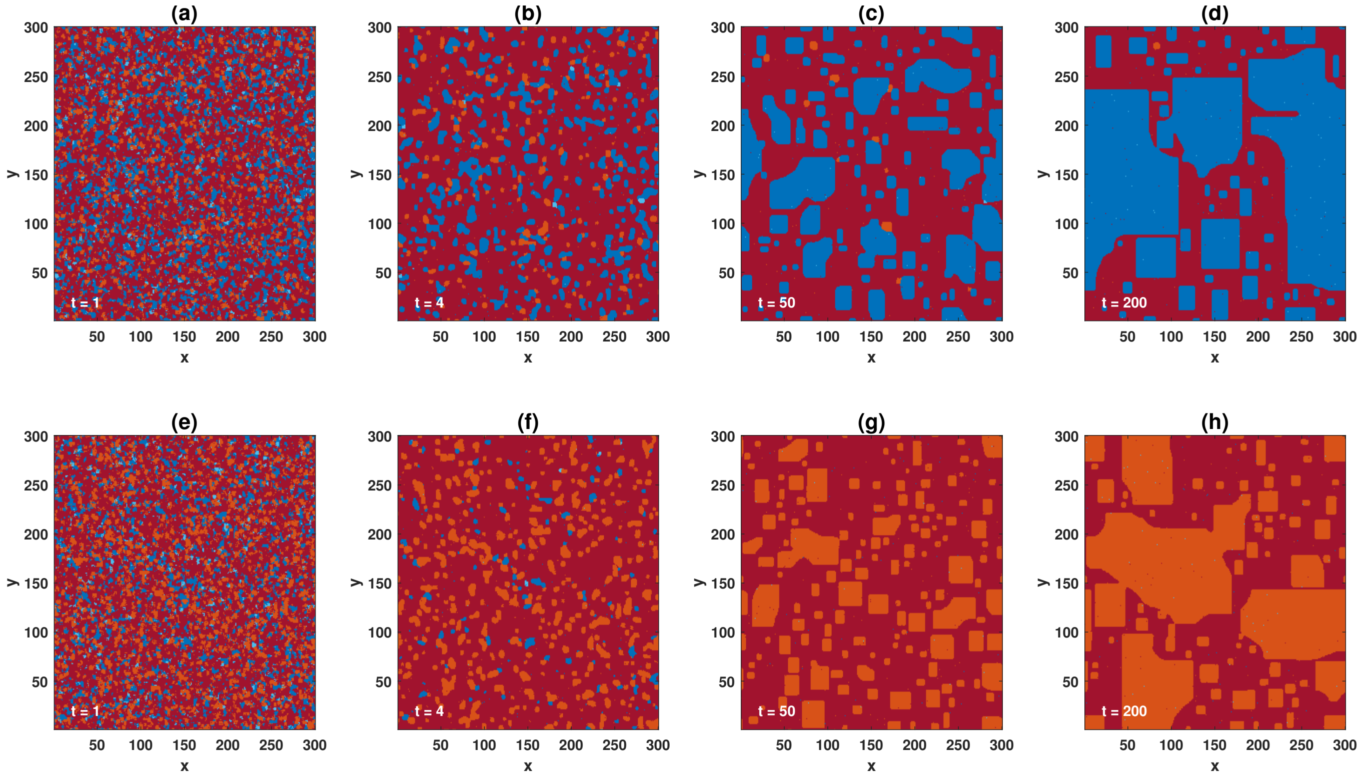

FIG. S.13. Time evolution of the system, close to the $PD-PC$ phase transition ((a) to (d)), and close to the $D-PD$ phase transition ((e) to (h)). Different strategies are indicated by different colors. Light blue shows non-punishing cooperators, dark blue shows punishing cooperators, light red shows non-punishing defectors, and dark red shows punishing defectors. (a) to (d): Starting from a random initial condition, close to the $PD-PC$ transition, punishing defectors rapidly increase in frequency and dominate the population. While all the other strategies go to extinction, punishing cooperators can survive by forming compact rectangular domains. The second stage of the evolution is governed by slow growth of punishing cooperators domains along their boundaries. (e) to (h): Close to the $D-PD$ transition, starting from a random initial condition, punishing defectors rapidly expand by driving all the other strategies into extinction. Only non-punishing defectors can survive by forming small rectangular domains. These blocks slowly increase in size in the see of punishing defectors along their boundaries, until they expand the whole population. In both cases, a population of $N=90000$ individuals lives on a two dimensional 300×300 lattice with periodic boundaries and Moore connectivity. In (a) to (d) $r=1.9$ and $\rho=5$, and in (e) to (h), $r=1.2$ and $\rho=4$. In all the cases $\nu=10^{-3}$, and $c=c'=1$.

C phase. As can be seen in the Video, starting from a random initial condition, non-punishing defectors rapidly drive other strategies into extinction, as here, the return to investments in punishing pool is not strong enough to promote punishing strategies. However, although they rapidly go to extinction, but by helping small islands of non-punishing cooperators to be formed in a sea of non-punishing defectors, they leave their footprint on the time evolution of the system. Afterwards, the dynamics of the system is driven by growth and collapse of such non-punishing cooperator islands in the sea of non-punishing defectors. We note that, in the absence of punishing strategies, non-punishing cooperators would have been unable to form small islands starting from a random initial condition, and would have been unable to survive, for this value of r and network size. In this way, the very existence of punishing strategies can help the evolution of cooperation, even in a phase where such strategies can not survive.

In the SV.3 we present the dynamics of the system close to the $D-PC$ transition. Here, $r=2.2$ and $\rho=3.2$. As can be seen in the Video, starting from a random initial condition, domains of homogeneous strategies are formed rapidly. Interestingly, domains of non-punishing cooperators, can only survive if engulfed with punishing defectors, but rapidly go to extinction if in contact with non-punishing defectors. This is due to the fact that, as punishment enhancement factor is not large enough here, punishment imposes a large cost on the punishers which is not compensated by its return. This increases the fitness of non-punishing defectors with respect to punishing defectors. At the second stage of the evolution, the sea of non-punishing defectors grows and drives both the domains of punishing defectors and non-punishing cooperators into extinction. Only punishing cooperators, are in a high enough advantage with respect to non-punishing defectors to be able to survive in rectangular domains. This sets the stage for the third stage of the evolution, in which punishing cooperators begin to slowly grow along the horizontal and vertical boundaries.

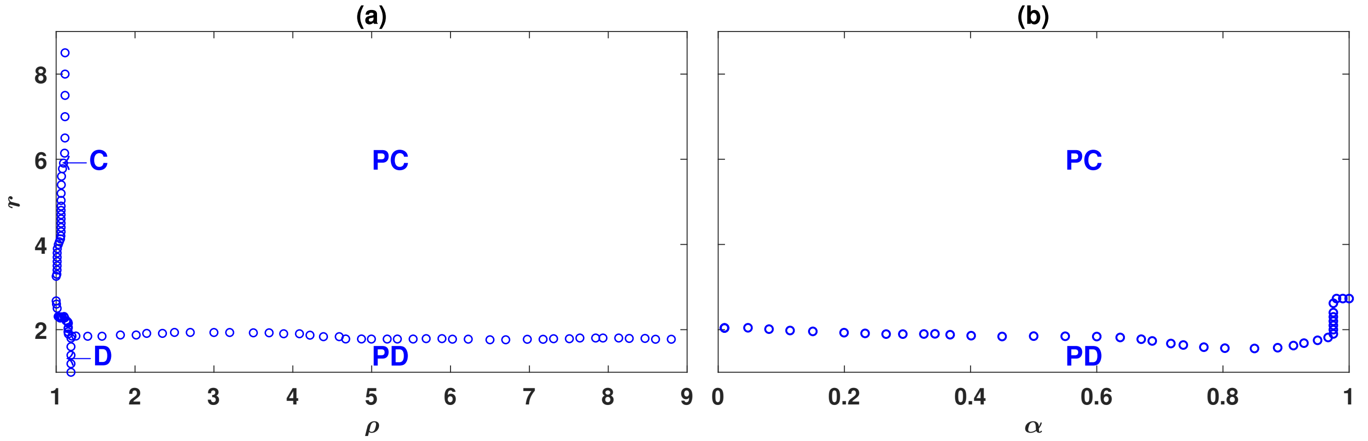

FIG. S.14. The phase diagram of the non-wasteful model for a structured population. The phase diagram is derived by running simulations in a population of size 160000, residing on a 400×400 first nearest neighbor lattice with Moore connectivity and periodic boundaries. Depending on the parameters of the model, the model shows four different phases. C, D, PC, and PD denote different phases in which, respectively, only, cooperators, defectors, punishing cooperators, and punishing defectors survive. Here, $g = 9$, $\nu = 0.001$, and $c = c' = 1$. In (a) $\alpha = 0.5$ and in (b) $\rho = 5$.

Interestingly, when two or more blocks come into contact due to slow growth along their boundaries, the domain of punishing cooperators rapidly grows until it takes a rectangular shape once again. This phenomenon results from the fact that, the growth of punishing cooperators is slowed along a horizontal or vertical boundary due to the fact that the amount of punishment a non-punishing defector receives from its neighboring punishing cooperators nearly offsets the advantage it receives due to not contributing to the public pool. However, as the number of neighboring punishing cooperators of a non-punishing defector increases, as it happens when the boundary of cooperators' domains is not a horizontal or vertical line, the amount of punishment a defector receives from punishing cooperators overcomes the gain the defector receives due to not contributing to the public good, by a large margin. Consequently, the speed of growth of punishing cooperators increases along such boundaries.

As mentioned before, the growth pattern described in the preceding paragraph possesses two different time scales. A slow time scale due to slow growth along the horizontal or vertical boundaries, and a fast time scale, due to rapid growth along other boundaries. As shown before, these two time scales lead to a staircase appearance of the time series of densities of different strategies, in which short periods of rapid growth are intermittent between long time intervals of slow growth. Such a phenomenology, is generally observed close to all the first order transitions of the system.

In SV.4 we present the dynamics of the system close to the $D - PD$ transition. Here, $r = 1.2$ and $\rho = 4$. These values are chosen such that the system is in the D phase, close to the $D - PD$ phase transition. As can be seen in the video, punishing defectors rapidly drive all the strategies into extinction. However, small domains of non-punishing defectors are formed in a sea of punishing defectors. As was the case, for other transitions, these blocks of non-punishing defectors start to grow along the boundaries. Furthermore, their growth shows tow different time scales. A small growth along the horizontal and vertical boundaries, intermittent with a rapid growth when different blocks collide.

Finally, in SV.5, we have set $r = 1.9$ and $\rho = 5$, such that the system is posed close to the $PC - PD$ transition, in the PC phase. the same growth pattern observed in the other cases is at work here. Punishing defectors rapidly drive non-punishing defectors and non-punishing cooperators into extinction. Consequently, a sea of punishing defectors is formed in which all the other strategies can survive by forming homogeneous domains. Among these, only punishing cooperators are in a large enough advantage with respect to punishing defectors, to grow in the sea. Consequently, domains of punishing cooperators start to grow until they take over the whole population. As before, here the growth pattern of punishing cooperators show two different time scales: a slow growth along the horizontal and vertical boundaries, intermittent by short periods of fast growth when rectangular blocks of punishing cooperators collide, resulting in non-horizontal and non-vertical boundaries.

S. 6. NON-WASTEFUL PUNISHMENT IN A STRUCTURED POPULATION

We present the phase diagram of the non-wasteful punishment model in the case of a structured population in Fig. (S.14). Here, simulations are performed in a population of size $N = 160000$ individuals residing on a 400×400 first nearest neighbor two dimensional square lattice, with Moore connectivity and periodic boundaries. In Fig. (S.14.a) we have set $g = 9$, $\nu = 10^{-3}$, and $\alpha = 0.5$, and in Fig. (S.14.b), we have set $g = 9$, $\nu = 10^{-3}$, and $\rho = 5$.

The non-wasteful punishment model in a structured population shows similar phases to those observed in the wasteful punishment model. However, there are interesting differences with the wasteful punishment model. First, for a fixed r, the phase transition to a phase where punishing institutions evolve shifts to smaller punishment enhancement factors. In contrast, the transition from antisocial punishment to social punishment for a fixed ρ does not show significant difference in the non-wasteful punishment model, compared to the wasteful punishment model. We note that, the transition to the evolution of punishing institution occurs for a smaller value of ρ in a structured population, compared to that in the case of a well-mixed population. This shows that when punishment is non-wasteful, it evolves with more ease in a structured population compared to a mixed population. This contrasts the situation in the wasteful punishment model where, as we saw, network structure could hinder the evolution of punishing institutions.

Interestingly, the transition from the D phase to the C phase is lost in the non-wasteful punishment model. Instead, by increasing r for a small ρ, the system shows a phase transition from the D phase to the PC phase. Further increasing r, the system shows a phase transition from the PC phase to the C phase. This phenomenology suggest that, just like the wasteful punishment model, the evolution of social punishment is facilitated close to the $C - D$ phase transition as well, as in this region, social punishment evolves for smaller returns to the investments in the punishment pool, ρ.

The phase diagram in the $\alpha - r$ plane shows that, contrary to the wasteful punishment model, second order punishment is not necessary for the evolution of punishment in this case: For the chosen value of ρ, punishing institutes evolve for all the values of α. For smaller r, antisocial punishment evolves, as r increases, social punishment evolves. As we saw, this was the case in a well-mixed population as well.
