# Supplementary material for: Evolution of prosocial punishment in unstructured and structured populations and in the presence of antisocial punishment
Source: PLoS One. 2021 Aug 6;16(8):e0254860. doi: 10.1371/journal.pone.0254860 (PMC8345862; doi:10.1371/journal.pone.0254860)
Supplement: Attachment — Submitted filename: response.pdf [file pone.0254860.s007.pdf]

Responses to review comments

Response to Reviewer N. 1

Reviewer N. 1:
In this manuscript, the author has investigated the coevolution of social punishment and antisocial punishment in the public goods game. By using numerical calculations and simulations, they find that there exists an optimal weight for second-order that facilitates the evolution of cooperation. Furthermore, the author has extended the model to structured populations, and found that the mechanism is further strengthened in a cooperation favoring environment. The motivation of this manuscript is clear and the idea is novel. Thus I recommend the publication of this meaningful manuscript after some minor corrections detailed below:
Response:
I am thankful to the reviewer for his/her careful reading and assessment of the manuscript and for and for his/her constructive suggestions, and for finding the manuscript of interest.
Reviewer N. 1:
1. In the Model section, the author uses the term anti-social punishment. As a corresponding, I suggest that the author change social into pro-social (line 61).
Response:
I have applied this suggestion.
Reviewer N. 1:
2. I suggest that the author change the C, D, PC and PD in Figures 1 and 2 to italics.
Response:
This suggestion is applied in the revision.
Reviewer N. 1:
3. The author analyzes the replication dynamics by used numerical calculation and simulations, so I suggest the author change the title "The simulations and analytical solutions" to "The simulations and numerical solutions" (line 287).
Response:
I have applied this suggestion.
Reviewer N. 1:
4. In introduction section, the authors mentioned that altruistic punishment can play an important role in the evolution of cooperation. However, there are still some important literatures have not been reviewed, such as PLoS ONE, 16 (1), e0244592; New Journal of Physics, 16 (8), 083016; Proceedings of the Thirtieth AAAI Conference on Artificial Intelligence. 2016: 2494-2500;.Journal of the Royal Society Interface, 12 (102), 20140935; Mathematical Models and Methods in Applied Sciences, 29 (11), 2127-2149. These literatures are very relevant to the subject of punishment and cooperation.
Response:
I have used these suggestions in the revised version to improve reference to the literature.
Reviewer N. 1:
5. The reference format should be adjusted to meet the requirements of PLoS ONE.
Response:
The reference format is updated.

Response to Reviewer N. 2

Reviewer N. 2:
The author studied how the punishing institution can evolve with cooperation in the presence of an antisocial punishment strategy. The results showed that punishing cooperators (who investigate both public and punishment pools) can evolve even without cooperation favoring mechanisms when the punishment enhancement factor is high enough and the institution punishes both defectors and the second-order free riders in an appropriate ratio. It implies that punishing both the first-order and the second-order free-riders is essential for the evolution of the punishing institution. The model explanation is clear, and the paper is well organized. However, there are some places to be improved for better readability. So, I recommend the publication of the manuscript in PLOS ONE after a minor revision.
Response:
I am thankful to the reviewer for his/her careful reading and assessment of the manuscript and for and for his/her constructive suggestions and for finding the manuscript acceptable.
Reviewer N. 2:
[minor suggestions] 1. In the abstract and introduction, "general environment" sounds unclear. It would be better to describe it specifically (e.g., environments with cooperation favoring mechanisms).
Response:
General environment is defined as a context where a cooperation favoring mechanism is not at work. I have removed this term in the abstract and described it in the introduction.
Reviewer N. 2:
2. In the last sentence of the first paragraph on page 1, the author said "However, the important question that whether altruistic punishment on its own can promote cooperation has remained unaddressed." But I am not sure that the main text can answer this. Did you mention it because the transition from PD to PC happens much lower r-value than the transition from D to C? If so, it would be good to highlight it.
Response:
Yes, this is the case because the transition to the PC phase occurs for r smaller than that for the transition from D to C.
Reviewer N. 2:
3. Fig. S.4. is explained before Fig. S.3. It would be better to change the order of figures.

Response:
The order of figures is changed to conform with the text.

Response to Reviewer N. 3

Reviewer N. 3:
The manuscript presents a model of evolution of cooperation considering pool punishment. The model considers the evolution of both punisher cooperators and defectors doing antisocial punishment. The main contribution of this model is to show that under the right conditions, cooperation and punishment can evolve without requiring other mechanisms favouring cooperation. Overall, the paper is well written, the goal and the contribution are well presented. The model used is straightforward, easy to understand and adapted to answer the research question. The results are well presented, with the author using phase transition plots to provide a global view of the results. I am in favour of accepting the manuscript with minor modifications.
Response:

I am thankful to the reviewer for his/her careful reading and assessment of the manuscript, and for his/her constructive suggestions.

Reviewer N. 3:

My main comment is that the manuscript could be improved by explaining clearly why the model presented obtains cooperation using pool punishment when previous models did not. For instance, in the paper of (Sigmund et al. 2010), they do not observe the evolution of punishment and cooperation when they consider that the participation to pool punishment is compulsory. Yet, they appear to have a similar model than the one presented here. Why does the model presented here find different results? Is it because the author explores a larger parameter space, e.g. high punishment enhancement factor? Or is it because the group size is fixed to a low value of 9? Explaining this difference of results would help the claim of the manuscript to get accepted by a reader, in particular as the novelty of contributions in social evolution are often debated (Kay, Keller, and Lehmann 2020).
On the same note, the authors also claims that it introduces a public punishing game where individuals can pay a fee to an institution that punish both free-riders not contributing to the public good game and free-riders not contributing to the punishment pool. However, I struggle to see the difference between this public punishing game and pool-punishment systems introduced in previous models , such as in (Perc 2012; Sigmund et al. 2010). If there are no fundamental differences, the author needs to cite these previous work. If there are differences, the author should make them clear in the manuscript.

Response:

I have discussed these works in the revised manuscript and have clarified the difference of the model introduced here with the past models of pool punishment. As discussed in the manuscript, punishment pool in my manuscript is a public goods game in the theoretical sense: Its resources is equal to an enhancement factor ρ times the sum of all the contribution to the punishment pool. In past models of pool punishment this is not the case. The difference between this model and previous models can be part of explanation why past work have not detected the evolution of punishment. Not exploring a broad parameter regime can be another reason. Although, due to the difference between this model and the model of Sigmund et al. 2010, where the authors specify their model does not give rise to punishment in a mixed population, comparison of parameters is not really possible or straight forward: While it seems safe to say their model is confined to $\alpha = 0.5$, on the basis of which it can be argued they have not explored a large parameter regime, ρ in this model has completely different meanings compared to B and G in their model. Besides, the nature of the evolutionary dynamics used is different in their work (the authors use a pairwise imitation process in a finite population), which makes comparison more difficult. Similar differences in models hold with other works such as Perc 2012.

Reviewer N. 3:

The supplementary material is very long and I am not sure it is necessary to include all these information (I have read it but I did not proofread it in details). It is not very clear to me the interest of characterising the transitions and I do not know of a community with an interest on this particular topic. If I am not mistaken, the author could cut down the supplementary materials.

Response:

I have kept this analysis in the Supplementary Material. I understand that the study of phase transitions might not be of interest to the majority of the readers. However, it does not seem keeping this analysis in the supplementary information to be hurtful.

Reviewer N. 3:

A list of minor comments: I recommend changing the title because institutions are not truly evolving in this model.

Response:

I have changed the title of the manuscript.

Reviewer N. 3:

Abstract: Nevertheless, theoretical work has been unable to show how this is possible. . This is not exactly true (as the author explains after) and it downplays a lot of theoretical work. I suggest removing it.

Response:

I have removed this sentence.

Reviewer N. 3:

L29: Remove its exclusion is a point which a proper theory needs to address.. This sounds like previous theory were not proper.

Response:

I have removed the term "proper", and have added more explanation.

Reviewer N. 3:

L62: What would happen if the amount invested in social punishment can differ from the amount invested in antisocial punishment?

Response:

I have added a figure to the manuscript to address this question. For too small c' (compared to c) antisocial punishment is favored, in the sense that it evolves for a larger set of parameter values. And for too large c' punishment (prosocial or antisocial) does not evolve. So the most favorable condition for the evolution of prosocial punishment is provided when the values of c' and c are comparable.

Reviewer N. 3:

Add a sentence in the model definition that says that the extended description of the method can be found in supplementary material.

Response:

In the revised version, a comparison of the model with other models of pool punishment is added based on the Reviewer's comments. A virtue of this model is that conservation of wealth in the punishment pool can be taken into account and this provided new ways to extend the model, for instance by using the unused resources in punishment pool for rewarding to (prosocial or antisocial) punishers. This point appears to be important enough to deserve to be paid more attention to in the main text, and thus I have brought more discussion about the extended model into the main text (model section and result section).

Reviewer N. 3:

I think the most common term to describe the proportion of individual with a given strategy is frequency instead of density.

Response:

I have changed density to frequency.

Reviewer N. 3:

L125: Why do the simulations not always use the same population size? There are results with population size of 10 000, 40 000 and 90 000.

Response:

This allows us to study the effect of population size and make sure the results are valid for different population sizes. This is also of interest in the study of phase transitions.

Reviewer N. 3:

L137: I am not sure this result comes (exclusively) from the rock-paper-scissor dynamic. Are not punishers rare for this transition because the punishment enhancement factor is low? Is this result not coming from the spatial structure which is known to favour the evolution of cooperation?

Response:

Although rare in equilibrium, punishers facilitate the dominance of non-punishing cooperators in the

initial stage of the evolution. After they exclude defectors, non-punishing cooperators are in advantage and grow in the sea of punishing cooperators, and thus drive the latter into extinction. This does not come exclusively from the rock-paper-scissor like dynamics (as it is absent in a mixed population, and the possibility of forming homogeneous blocks is essential) and comes from the synergistic role of this dynamics and spatial structure. I have modified this part to make this point clear.

Reviewer N. 3:

L180: This implies that all the transitions but the D - C transition are discontinuous. What does a discontinuous transition mean exactly?

Response:

In a discontinuous transition the system is bistable (or multi-stable) close to the transition region, and the equilibrium state depends on the initial condition. For this reason I have mentioned bistability implies a discontinuous phase transition. In a discontinuous phase transition, the order parameter (here, for example ρ_C) of the system changes abruptly as a function of the control parameter (here r or ρ) when passing the phase transition point. On the other hand in a continuous phase transition the system is not bistable, and the order parameter changes continuously as a function of the control parameter (here r)

Reviewer N. 3:

L201: It also reveals network structure can be detrimental to the evolution of punishing institutions. Is it truly the case? Yes, the punishment enhancement factor needs to be slightly higher, but the public good enhancement factor can be much lower (2 instead of 9).

Response:

In this phrase I had used "punishing institution" and meant punishing institutions in general, be it prosocial (PC) or antisocial (PD). In this sense, network structure hinders the evolution of punishing institutions. Yet, it is also true that is facilitates the evolution of prosocial as opposed to antisocial punishment as it shifts PC-PD transition to smaller values. So, in this sense, it has also beneficial effect for the evolution of prosocial punishment. I have modified this part of manuscript to make this point clear.

Reviewer N. 3:

L205: Finally, we have seen that being close to a physical phase transition is beneficial for the evolution of punishing institutions. Is this the case only in the spatial model? If yes, specify it.

Response:

This seems to also hold in a mixed population. However, the shift is very small in a mixed population. I have specified that this holds in a structured population.

Reviewer N. 3:

L211: public punishing game introduced here, Again, it is not clear in which way the public punishing game of the manuscript is new. It looks similar to previous pool punishment models.

Response:

This is explained in the manuscript now.

Reviewer N. 3:

The model presented in (Perc 2012) is very similar to the spatial model in this manuscript. Could they be quickly compared in the discussion?

Response:

Both Sigmund et al. 2010, and Perc 2012 are discussed in the Model section, and mentioned in the introduction and discussion

Reviewer N. 3:

An important parameter in the model is the punishment enhancement factor and the range of value considered can be high (up to 9). Is there a reason to think that such value would exist in real world? If yes, could the author provide an example?

Response:
To my knowledge the most common value for punishment enhancement factor, at least in peer punishment models, is 3 (for instance in public goods with punishment experiments (Chaudhuri, A., 2011. Sustaining cooperation in laboratory public goods experiments: a selective survey of the literature. Experimental economics, 14(1), pp.47-83.)). The model predicts prosocial punishment can evolve already for values close to 3. As for larger values, it seems to me such values can be plausible. For instance, depriving a piece of bread from an starving individual could be a matter of life an death. Besides, in this model, punishment pool is considered as a public goods game, and ρ can be considered as the enhancement factor of a public goods game. As, usually enhancement factor as large as the groups size are studied, it seems useful to study the behavior of the model for large values of ρ. For instance, one of the findings of the model is that larger punishment enhancement factors facilitate the evolution of prosocial instead of antisocial punishment. Studying larger values of ρ allows to see this result hold for a broad range of values of ρ.

Reviewer N. 3:
Supplementary materials S3.1 L4: a e is missing in th

Response:
This is corrected.

Reviewer N. 3:
Bibrliography: Kay, Tomas, Laurent Keller, and Laurent Lehmann. 2020. The Evolution of Altruism and the Serial Rediscovery of the Role of Relatedness. Proceedings of the National Academy of Sciences, 202013596. Perc, Matja. 2012. Sustainable Institutionalized Punishment Requires Elimination of Second-Order Free-Riders. Scientific Reports. Sigmund, Karl, Hannelore De Silva, Arne Traulsen, and Christoph Hauert. 2010. Social Learning Promotes Institutions for Governing the Commons. Nature 466 (7308): 86163.
